# Gasdermin D independent canonical inflammasome responses cooperate with caspase-8 to establish host defense against gastrointestinal *Citrobacter rodentium* infection

**DOI:** 10.1038/s41419-023-05801-4

**Published:** 2023-04-21

**Authors:** Elien Eeckhout, Lisa Hamerlinck, Veronique Jonckheere, Petra Van Damme, Geert van Loo, Andy Wullaert

**Affiliations:** 1 Department of Internal Medicine and Paediatrics, Ghent University, Ghent, Belgium; 2 VIB-UGent Center for Inflammation Research, VIB, Ghent, Belgium; 3 iRIP Unit, Laboratory of Microbiology, Department of Biochemistry and Microbiology, Ghent University, Ghent, Belgium; 4 Department of Biomedical Molecular Biology, Ghent University, Ghent, Belgium; 5 Laboratory of Proteinscience, Proteomics and Epigenetic Signalling (PPES), Department of Biomedical Sciences, University of Antwerp, Antwerp, Belgium

**Keywords:** Immune cell death, Infection, Signal transduction, Cell death

## Abstract

*Citrobacter rodentium* is an enteropathogen that causes intestinal inflammatory responses in mice reminiscent of the pathology provoked by enteropathogenic and enterohemorrhagic *Escherichia coli* infections in humans. *C. rodentium* expresses various virulence factors that target specific signaling proteins involved in executing apoptotic, necroptotic and pyroptotic cell death, suggesting that each of these distinct cell death modes performs essential host defense functions that the pathogen aims to disturb. However, the relative contributions of apoptosis, necroptosis and pyroptosis in protecting the host against *C. rodentium* have not been elucidated. Here we used mice with single or combined deficiencies in essential signaling proteins controlling apoptotic, necroptotic or pyroptotic cell death to reveal the roles of these cell death modes in host defense against *C. rodentium*. Gastrointestinal *C. rodentium* infections in mice lacking GSDMD and/or MLKL showed that both pyroptosis and necroptosis were dispensable for pathogen clearance. In contrast, while RIPK3-deficient mice showed normal *C. rodentium* clearance, mice with combined caspase-8 and RIPK3 deficiencies failed to clear intestinal pathogen loads. Although this demonstrated a crucial role for caspase-8 signaling in establishing intestinal host defense, Casp8^–/–^Ripk3^–/–^ mice remained capable of preventing systemic pathogen persistence. This systemic host defense relied on inflammasome signaling, as Casp8^–/–^Ripk3^–/–^ mice with combined caspase-1 and -11 deletion succumbed to *C. rodentium* infection. Interestingly, although it is known that *C. rodentium* can activate the non-canonical caspase-11 inflammasome, selectively disabling canonical inflammasome signaling by single caspase-1 deletion sufficed to render Casp8^–/–^Ripk3^–/–^ mice vulnerable to *C. rodentium*-induced lethality. Moreover, Casp8^–/–^Ripk3^–/–^ mice lacking GSDMD survived a *C. rodentium* infection, suggesting that pyroptosis was not crucial for the protective functions of canonical inflammasomes in these mice. Taken together, our mouse genetic experiments revealed an essential cooperation between caspase-8 signaling and GSDMD-independent canonical inflammasome signaling to establish intestinal and systemic host defense against gastrointestinal *C. rodentium* infection.

## Introduction

During a bacterial infection, microbial ligands as well as host-derived cytokines can trigger signaling pathways that control the execution of functionally distinct forms of programmed cell death (PCD) such as apoptosis, necroptosis and pyroptosis. These PCD modes differentially regulate host immune responses as well as pathogen survival. For instance, non-lytic apoptosis may serve as an immunologically silent way to eliminate the replicative niche of intracellular pathogens, while lytic forms of PCD such as necroptosis and pyroptosis control infections by initiating inflammation and recruiting specialized effector immune cells [1, 2]. As a countermeasure to these host PCD responses, pathogens evolved various virulence factors to curb PCD signaling pathways to their advantage, which in turn inspired the host to establish signaling redundancies and back-up PCD pathways. The result is a complex signaling network in which the type of PCD outcome and the eventual impact on host defense depend on the nature of the pathogen as well as on the host cell type infected [1, 2]. Therefore, for any given pathogen it is important to disentangle the relative contributions and interplays of various PCD modes to fully understand how the immune response achieves host defense against that pathogen.

*Citrobacter rodentium* is a Gram-negative extracellular enteropathogen that causes attaching and effacing lesions leading to a self-limiting colon inflammation in mice, which is a widely used model to study the pathogenesis of closely related enteropathogenic *E. coli* (EPEC) and enterohaemorrhagic *E. coli* (EHEC) human pathogens [3]. Several studies identified PCD-interfering *C. rodentium* virulence factors suggesting that apoptotic, necroptotic as well as pyroptotic cell death may contribute to host defense [4]. For instance, *C. rodentium* expresses NleB that glycosylates several death domain proteins in caspase-8 activating death receptor complexes and thereby blocks both apoptosis and necroptosis [5, 6]. Ablating NleB reduced early colonic *C. rodentium* loads, suggesting that death receptor signaling to apoptosis and necroptosis contributes to limiting pathogen colonization [5–8]. Interestingly, deficiency in the cysteine protease EspL that cleaves RIPK1 and thus specifically blocks necroptosis attenuated *C. rodentium* loads in the resolving phase of the infection, suggesting that necroptosis rather contributes to pathogen clearance [9]. Additionally, *C. rodentium* expresses NleF that inhibits caspase-11 activity and can thereby block non-canonical inflammasome signaling to pyroptosis [10]. Although NleF deficiency did not alter in vivo *C. rodentium* virulence [10, 11], NleA-deficient *C. rodentium* were severely attenuated [12]. As NleA of closely related EPEC inhibits Nlrp3 inflammasome signaling [13], these bacterial genetic studies suggested that canonical rather than non-canonical inflammasome signaling prevails in host defense against *C. rodentium*. Host genetic studies supported this notion, as double caspase-1/11 deficient mice were hypersusceptible to *C. rodentium* while single caspase-11 deficient mice only showed moderately elevated pathogen shedding [14, 15]. However, the potential contribution of downstream pyroptosis in these inflammasome effects remains unknown, since the role of the pore-forming pyroptosis executor Gasdermin D (GSDMD) in *C. rodentium* infection has not been studied. Likewise, despite the NleB and EspL studies suggesting necroptosis roles in constraining *C. rodentium* [5–7, 9], also the role of the pore-forming necroptosis executor mixed lineage kinase domain like (MLKL) has not been studied. Finally, although caspase-8 deficiency leads to higher *C. rodentium* shedding at 15 days post-infection [16], it is not known how the innate immune system guarantees host defense against *C. rodentium* in conditions of impaired caspase-8 signaling.

Here, we compared the relative contributions of GSDMD-, MLKL- and caspase-8-mediated signaling to host defense against gastrointestinal *C. rodentium* infection. We show that caspase-8 is needed for intestinal pathogen control, as caspase-8 deficient mice failed to clear colonic *C. rodentium* loads. Interestingly, GSDMD-independent canonical inflammasome responses were required to prevent progress to a lethal systemic infection in these mice. Thus, our observations reveal a crucial cooperativity between caspase-8 and inflammasome signaling pathways to establish effective host defense against gastrointestinal *C. rodentium* infection.

## Results

### GSDMD-mediated pyroptosis does not critically contribute to *C. rodentium* host defense

**Fig. 1:**
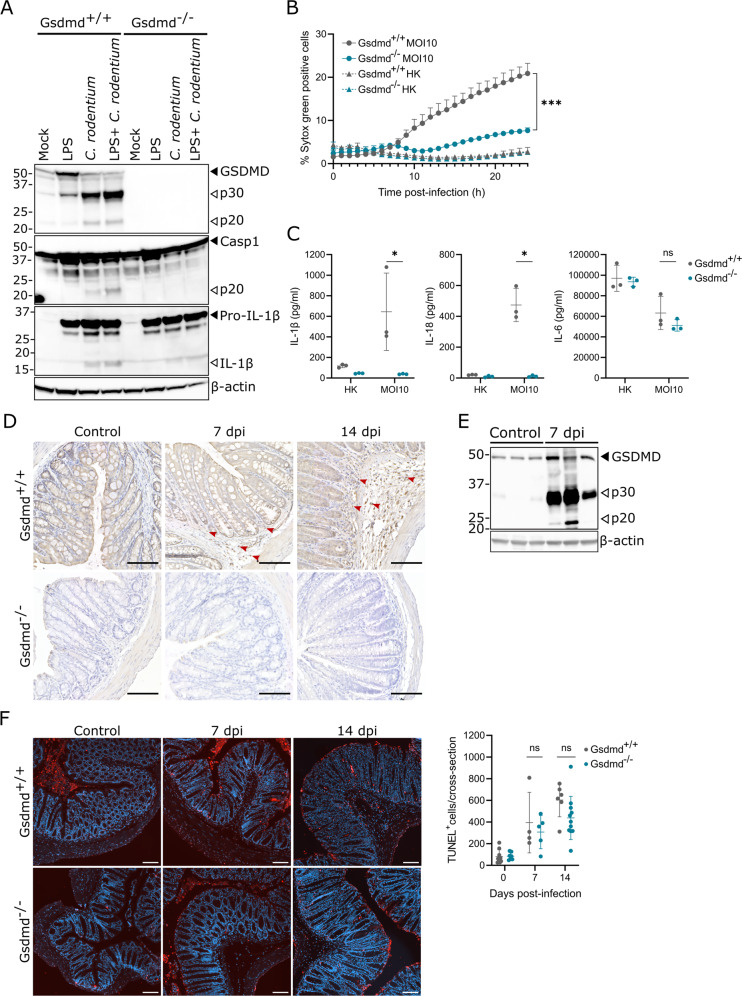
*C. rodentium* induces GSDMD-dependent pyroptosis in macrophages but GSDMD-independent cell death in IECs. **A** Western blot analyses of Gsdmd^+/+^ and Gsdmd^–/–^ BMDMs collected after 24 h of indicated treatments. Results shown are representative of two independent experiments. **B** Realtime cell membrane permeability analysis of Gsdmd^+/+^ and Gsdmd^–/–^ BMDMs either infected with live *C. rodentium* (MOI 10) or provided with equal amounts of heat-killed (HK) *C. rodentium*. Data are means + SD of biological triplicates. **C** IL-1β, IL-18, and IL-6 measurements in culture supernatant of Gsdmd^+/+^ and Gsdmd^–/–^ BMDMs either infected with live *C. rodentium* (MOI 10) or provided with equal amounts of heat-killed (HK) *C. rodentium* for 24 h. Data are means ± SD of biological triplicates. **D** Representative colon GSDMD IHC stainings from Gsdmd^+/+^ and Gsdmd^–/–^ littermates not infected or infected with 5 × 10^9^ CFU *C. rodentium* for 7 or 14 days. Red arrowheads show examples of GSDMD^+^ non-epithelial cells in infected Gsdmd^+/+^ mice. Scale bars 100 µm. **E** Western blot analyses on whole colon lysates from WT mice not infected or infected with 5 × 10^9^ CFU *C. rodentium* for 7 days. Every lane represents a whole colon lysate from a different mouse. **F** Representative colon TUNEL stainings and quantifications from Gsdmd^+/+^ and Gsdmd^–/–^ littermates not infected or infected with 5 × 10^9^ CFU *C. rodentium* for 7 or 14 days. Every data point in the quantification represents a different mouse with means ± SD, *n* = 4–11 per group. Scale bars 100 µm.

In macrophages, *C. rodentium* triggers the non-canonical inflammasome pathway to pyroptosis, in which lipopolysaccharide (LPS) delivered to the cytosol by outer membrane vesicles activates caspase-11 that then cleaves GSDMD [17–20]. Subsequent pore formation by GSDMD elicits pyroptosis but also allows ion fluxes that activate the Nlrp3 inflammasome, resulting in caspase-1 mediated maturation and subsequent secretion of Interleukin (IL)-1β and IL-18 [21–23]. We first validated these inflammasome responses by infecting Gsdmd^+/+^ and Gsdmd^–/–^ bone marrow-derived macrophages (BMDMs) with *C. rodentium* without and with prior LPS stimulation, the latter to ensure proper TLR4-TRIF mediated priming of the non-canonical inflammasome [14, 24]. Western blotting analyses showed that Gsdmd^+/+^ BMDMs displayed cleavage of GSDMD, caspase-1 and pro-IL-1β in both infected conditions, whereas Gsdmd^–/–^ BMDMs showed impaired caspase-1 and pro-IL-1β processing (Fig. 1A). These results confirmed that *C. rodentium* activated the non-canonical inflammasome pathway in which GSDMD cleavage acts upstream of caspase-1-mediated IL-1β maturation, and indicated that TLR4 triggering by *C. rodentium* itself was sufficient to license activation of this pathway. We, therefore, used unprimed BMDMs in further infection experiments. Realtime cell membrane permeability imaging showed that GSDMD deficiency almost completely abrogated *C. rodentium*-induced cell death in BMDMs (Fig. 1B). Moreover, Gsdmd^–/–^ BMDMs failed to secrete IL-1β and IL-18 but released similar amounts of inflammasome-independent IL-6 upon *C. rodentium* infection compared to Gsdmd^+/+^ BMDMs (Fig. 1C). These results confirmed the essential role of GSDMD in *C. rodentium*-induced pyroptosis and ensuing IL-1β and IL-18 responses in macrophages. We next aimed to evaluate the function of GSDMD in *C. rodentium*-induced intestinal epithelial cell (IEC) death. Immunohistochemical (IHC) analyses showed that GSDMD was widely expressed in IECs of both naïve and infected mice, while infected mice displayed inflamed areas with non-epithelial GSDMD^+^ cells (Fig. 1D). Moreover, whole colon lysates from *C. rodentium*-infected mice at 7 days post-infection (dpi) displayed GSDMD cleavage (Fig. 1E). However, both at 7 and 14 dpi, Gsdmd^+/+^ and Gsdmd^–/–^ colons displayed similar numbers of TUNEL^+^ cells (Fig. 1F), suggesting that IEC death during *C. rodentium* infection was GSDMD-independent.

**Fig. 2:**
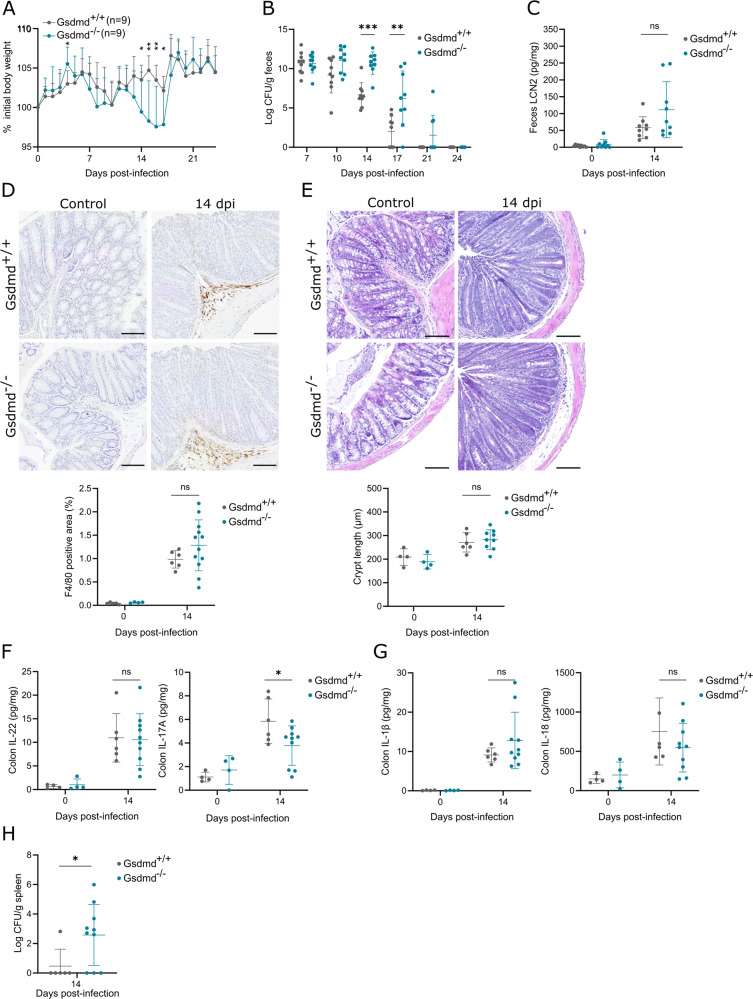
GSDMD deficiency transiently delays pathogen clearance but does not impair host defense against gastrointestinal *C. rodentium* infection. Age- and sex-matched Gsdmd^+/+^ (*n* *=* 9) and Gsdmd^–/–^ (*n* *=* 9) littermates were infected by oral gavage with 5 × 10^9^ CFU *C. rodentium*. **A** Weight change, **B** fecal *C. rodentium* loads, and **C** fecal LCN2 levels at indicated dpi are shown. Data in (**A**) represent means + SD; data in (**B**, **C**) represent individual mice and their means ± SD. Age- and sex-matched Gsdmd^+/+^ (*n* = 6) and Gsdmd^–/–^ (*n* = 10–12) littermates were infected by oral gavage with 5 × 10^9^ CFU *C. rodentium* and were sacrificed at 14 dpi, along with non-infected control Gsdmd^+/+^ (*n* = 4) and Gsdmd^–/–^ (*n* = 4) littermates. Representative colon (**D**) macrophage IHC and (**E**) H&E stainings with respective (**D**) quantifications and (**E**) colon crypt length measurements; (**F**) colon IL-22 and IL-17 levels; (**G**) colon IL-1β and IL-18 levels; and (**H**) splenic *C. rodentium* loads. All data points represent individual mice along with means ± SD. Scale bars (**D**) 100 µm.

Having established that GSDMD is involved in *C. rodentium*-induced PCD in BMDMs but not in vivo in IECs, we proceeded evaluating its physiological effects during a *C. rodentium* gastrointestinal infection. For this purpose, Gsdmd^+/+^ and Gsdmd^–/–^ littermates were challenged orally with *C. rodentium* and were monitored until pathogen loads in the feces were undetectable. During the infection, Gsdmd^–/–^ mice displayed a temporary but significant decrease in body weight around 14 dpi compared to Gsdmd^+/+^ littermates (Fig. 2A). Accordingly, Gsdmd^–/–^ mice showed more fecal shedding of *C. rodentium* at 14 and 17 dpi, although both Gsdmd^–/–^ and Gsdmd^+/+^ cohorts resolved the infection by 21-24 dpi (Fig. 2B). Therefore, as Gsdmd^–/–^ mice displayed transiently enhanced susceptibility to *C. rodentium* around 14 dpi, we evaluated the effect of GSDMD on colon inflammation at this stage of infection. Quantifying fecal lipocalin-2 (LCN2) levels as a sensitive marker of intestinal inflammation [25] revealed no difference between *C. rodentium*-infected Gsdmd^–/–^ and Gsdmd^+/+^ mice at 14 dpi (Fig. 2C). Further supporting similar inflammation levels in these cohorts, IHC macrophage analyses showed no differences in the numbers of these infiltrating immune cells in colons of *C. rodentium*-infected Gsdmd^–/–^ and Gsdmd^+/+^ mice at 14 dpi (Fig. 2D). In addition, while colon crypt hyperplasia is a characteristic histopathological feature of *C. rodentium* colitis, Gsdmd^–/–^ and Gsdmd^+/+^ cohorts displayed comparable crypt elongation at 14 dpi (Fig. 2E). We next investigated whether increased *C. rodentium* shedding in Gsdmd^–/–^ mice could be linked to insufficient colonic IL-22 or IL-17A production, as both of these cytokines are crucial for *C. rodentium* clearance [26, 27]. Indeed, although IL-22 levels were not different, *C. rodentium*-infected Gsdmd^–/–^ mice showed less colon IL-17A levels compared to Gsdmd^+/+^ mice (Fig. 2F), suggesting that dampened IL-17A responses at 14 dpi might underlie the higher intestinal pathogen loads around that time point. Interestingly, colonic IL-1β and IL-18 levels at 14 dpi were not affected by GSDMD deficiency (Fig. 2G), arguing that maturation of these cytokines in *C. rodentium*-infected colons happened upstream of GSDMD and thus was likely performed by canonical inflammasome signaling. Finally, to address whether increased *C. rodentium* fecal shedding in Gsdmd^–/–^ mice led to more systemic dissemination, we measured pathogen burdens in the spleen. Indeed, splenic *C. rodentium* loads were substantially higher in Gsdmd^–/–^ mice at 14 dpi (Fig. 2H). Taken together, while our observations indicated that loss of GSDMD transiently elevated intestinal *C. rodentium* loads leading to increased presence in the spleen, the unaltered levels of colon inflammation and the similar pathogen clearance kinetics in Gsdmd^+/+^ and Gsdmd^–/–^ cohorts showed that a gastrointestinal *C. rodentium* infection can be effectively controlled in the absence of GSDMD-driven pyroptosis.

### MLKL-mediated necroptosis does not critically contribute to *C. rodentium* host defense

**Fig. 3:**
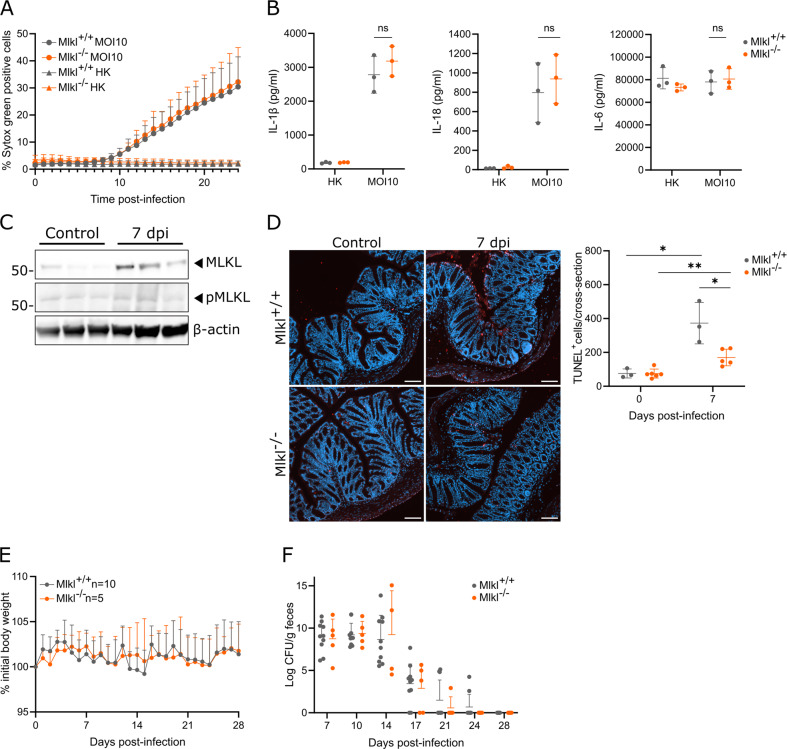
*C. rodentium* infection induces MLKL-dependent necroptosis in IECs but MLKL deficiency does not alter *C. rodentium* clearance kinetics. **A** Realtime cell membrane permeability analysis of Mlkl^+/+^ and Mlkl^–/–^ BMDMs either infected with live *C. rodentium* (MOI 10) or provided with equal amounts of heat-killed (HK) *C. rodentium*. Data are means + SD of biological triplicates. **B** IL-1β, IL-18, and IL-6 measurements in culture supernatants of Mlkl^+/+^ and Mlkl^–/–^ BMDMs either infected with live *C. rodentium* (MOI 10) or provided with equal amounts of heat-killed (HK) *C. rodentium* for 24 h. Data are means ± SD of biological triplicates. **C** Western blot analyses on whole colon lysates from WT mice not infected or infected with 5 × 10^9^ CFU *C. rodentium* for 7 days. Every lane represents a whole colon lysate from a different mouse. **D** Representative colon TUNEL stainings and quantifications from Mlkl^+/+^ and Mlkl^–/–^ littermates not infected or infected with 5 × 10^9^ CFU *C. rodentium* for 7 days. Every data point in the quantification represents a different mouse with means ± SD, n = 3-6 per group. Scale bars 100 µm. **E-F** Age- and sex-matched Mlkl^+/+^ (*n* = 10) and Mlkl^–/–^ (*n* = 5) littermates were infected by oral gavage with 5 × 10^9^ CFU *C. rodentium*. **E** Weight change, and **F** fecal *C. rodentium* loads at indicated dpi are shown. Data in (**E**) represent means + SD; data in (**F**) represent individual mice and their means ± SD.

Since GSDMD-driven pyroptosis was dispensable for *C. rodentium* host defense, we turned to investigating the role of necroptosis. Consistent with our above observation that *C. rodentium-induced* GSDMD-driven pyroptosis in BMDMs, disabling necroptosis through MLKL deficiency did not alter cell death kinetics in *C. rodentium*-infected BMDMs (Fig. 3A). Accordingly, also cytokine responses were similar in *C. rodentium*-infected Mlkl^+/+^ and Mlkl^–/–^ BMDMs (Fig. 3B). Western blotting analyses of whole colon lysates from *C. rodentium*-infected mice revealed increased MLKL expression at 7 dpi, while no alterations in MLKL phosphorylation could be detected (Fig. 3C). Nevertheless, quantifying TUNEL^+^ cells in *C. rodentium*-infected colons revealed that Mlkl^–/–^ mice harbored significantly less IEC death than their Mlkl^+/+^ littermates at 7 dpi, even though *C. rodentium*-infected Mlkl^–/–^ mice still showed elevated TUNEL^+^ cells in comparison with uninfected Mlkl^−/−^ controls (Fig. 3D). Thus, MLKL-driven necroptosis partially contributed to IEC death during an in vivo *C. rodentium* infection but was not involved in *C. rodentium*-induced PCD in BMDMs. We then evaluated the in vivo role of MLKL in *C. rodentium* host defense by monitoring orally infected Mlkl^+/+^ and Mlkl^−/−^ littermates. However, these cohorts displayed identical body weight loss and *C. rodentium* clearance kinetics (Fig. 3E, F), indicating no obvious role for MLKL-driven necroptosis in host defense against gastrointestinal *C. rodentium* infection.

**Fig. 4:**
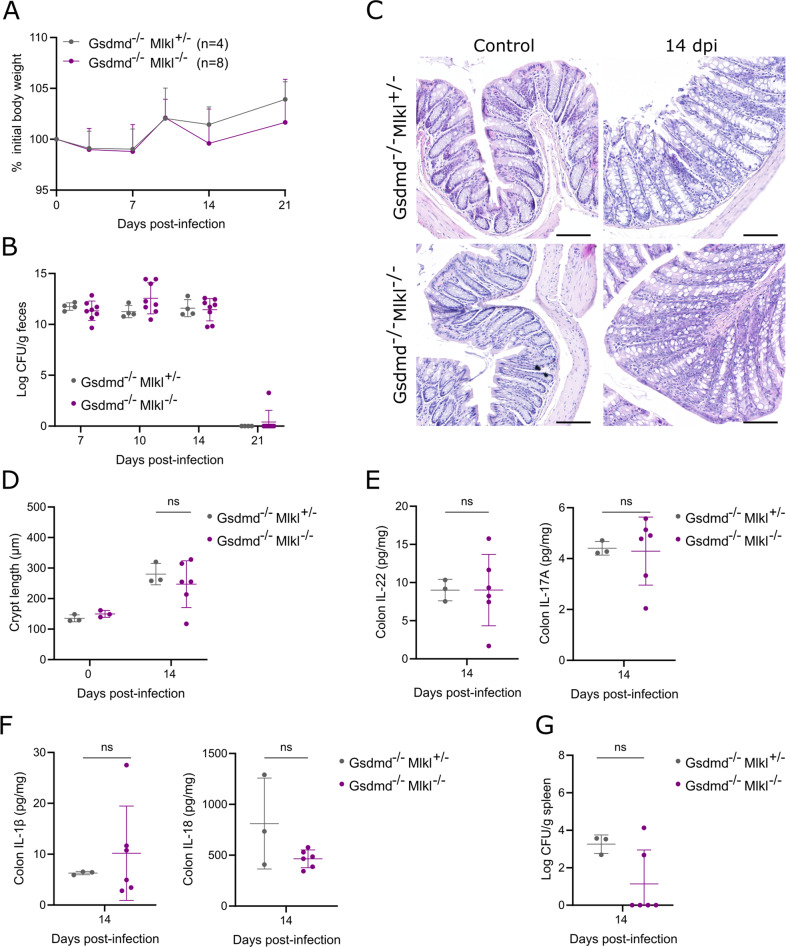
Combined GSDMD and MLKL deficiencies do not impair host defense against gastrointestinal *C. rodentium* infection. Age- and sex-matched Gsdmd^–/–^Mlkl^+/-^ (*n* = 4) and Gsdmd^–/–^Mlkl^–/–^ (*n* = 8) littermates were infected by oral gavage with 5 × 10^9^ CFU *C. rodentium*. **A** Weight change, and (**B**) fecal *C. rodentium* loads at indicated dpi are shown. Data in (**A**) represent means + SD; data in (**B**) represent individual mice and their means ± SD. (**C-G**) Age- and sex-matched Gsdmd^–/–^Mlkl^+/-^ (*n* = 3) and Gsdmd^–/–^Mlkl^–/–^ (*n* = 6) littermates were infected by oral gavage with 5 × 10^9^ CFU *C. rodentium* and were sacrificed at 14 dpi, along with non-infected control Gsdmd^–/–^Mlkl^+/-^ (*n* = 3) and Gsdmd^–/–^Mlkl^–/–^ (*n* = 3) littermates. **C** Representative colon H&E stainings and **D** colon crypt length measurements; **E** colon IL-22 and IL-17 levels; **F** colon IL-1β and IL-18 levels; and **G** splenic *C. rodentium* loads. All data points in (**D**–**G**) represent individual mice along with means ± SD. Scale bars (**C**) 100 µm.

Since our above observations showed that *C. rodentium* triggered GSDMD-driven pyroptosis in macrophages as well as MLKL-driven necroptosis in a subset of IECs during *C. rodentium* infection, we reasoned that these PCD responses acting in different cell types might cooperate to establish host defense against *C. rodentium*. To investigate this hypothesis, we generated Gsdmd^–/–^Mlkl^+/-^ and Gsdmd^–/–^Mlkl^–/–^ littermates allowing to evaluate whether MLKL deficiency aggravated the moderate *C. rodentium* susceptibility phenotype observed in Gsdmd^–/–^ mice. Monitoring body weight and fecal pathogen shedding however did not reveal differences between *C. rodentium*-infected Gsdmd^–/–^Mlkl^+/-^ and Gsdmd^–/–^Mlkl^–/–^ littermates (Fig. 4A, B). In addition, measuring crypt hyperplasia as well as quantifying colonic cytokine levels at 14 dpi showed that Gsdmd^–/–^Mlkl^+/-^ and Gsdmd^–/–^Mlkl^–/–^ littermates experienced similar colon inflammation at this stage of *C. rodentium* infection (Fig. 4C–F). Finally, splenic *C. rodentium* loads in Gsdmd^–/–^Mlkl^+/-^ mice at 14 dpi were at levels similar as observed earlier in infected Gsdmd^–/–^ mice, but were not further increased by additional MLKL deletion (Fig. 4G). In conclusion, these experiments using Gsdmd^–/–^, Mlkl^–/–^ as well as Gsdmd^–/–^Mlkl^–/–^ mice demonstrate that GSDMD-driven pyroptosis and MLKL-driven necroptosis do not exert separate or additive roles that are crucial for host defense against gastrointestinal *C. rodentium* infection.

### Caspase-8 signaling is crucial for preventing chronic intestinal *C. rodentium* infection but is dispensable for systemic clearance and host survival

**Fig. 5:**
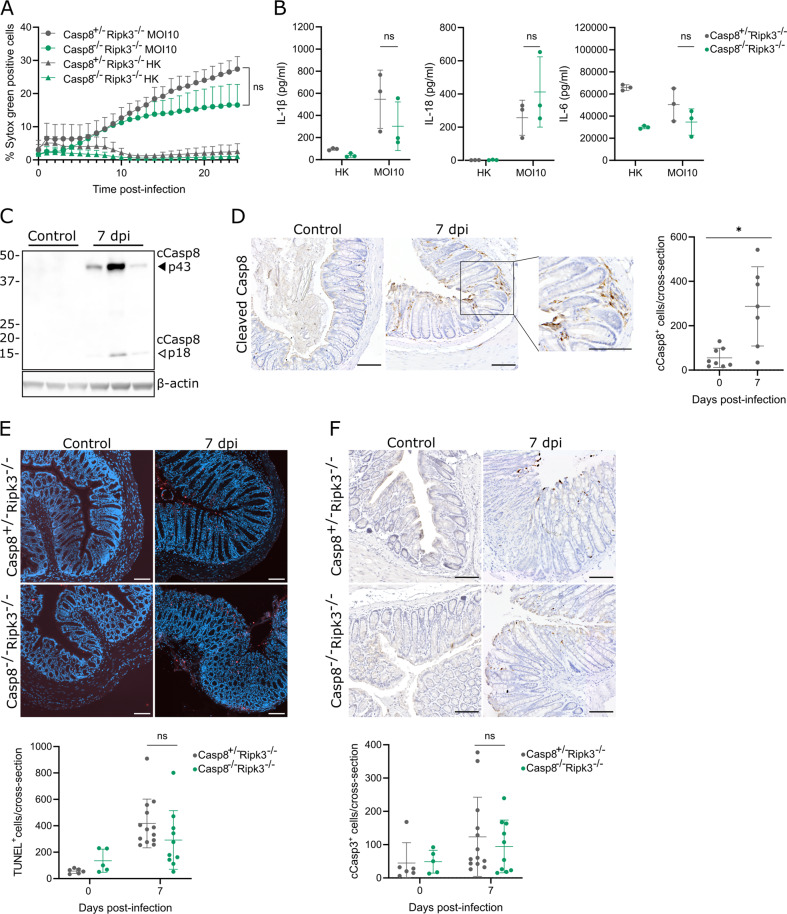
*C. rodentium* induces caspase-8-independent cell death in cultured macrophages, while *C. rodentium* infected mice display cCasp3-associated but caspase-8-independent colonic cell death. **A** Realtime cell membrane permeability analyses of Casp8^+/-^Ripk3^–/–^ and Casp8^–/–^Ripk3^–/–^ BMDMs either infected with live *C. rodentium* (MOI 10) or provided with equal amounts of heat-killed (HK) *C. rodentium*. Data are means + SD of biological triplicates. **B** IL-1β, IL-18, and IL-6 measurements in culture supernatant of Casp8^+/-^Ripk3^–/–^ and Casp8^–/–^Ripk3^–/–^ BMDMs either infected with live *C. rodentium* (MOI 10) or provided with equal amounts of heat-killed (HK) *C. rodentium* for 24 h. Data are means ± SD of biological triplicates. **C** Western blot analyses on whole colon lysates from WT mice not infected or infected with 5 × 10^9^ CFU *C. rodentium* for 7 days. Every lane represents a whole colon lysate from a different mouse. **D** Representative cCasp8 IHC staining on colons from WT mice not infected or infected with 5 × 10^9^ CFU *C. rodentium* for 7 days. Magnification shows area with both epithelial and non-epithelial cCasp8^+^ cells. Scale bars 100 µm. Representative colon (**E**) TUNEL and (**F**) cCasp3 stainings and respective quantifications from Casp8^+/-^Ripk3^–/–^ and Casp8^–/–^Ripk3^–/–^ littermates not infected or infected with 5 × 10^9^ CFU *C. rodentium* for 7 days. Every data point in the quantification represents a different mouse with means ± SD, *n* = 5-13 per group. Scale bars 100 µm.

To evaluate the role of apoptosis in *C. rodentium* host defense we used caspase-8 deficient mice on a Ripk3^–/–^ background to avoid embryonic lethality due to caspase-8 deficiency [28, 29]. Although these Casp8^–/–^Ripk3^–/–^ mice as well as their Casp8^+/-^Ripk3^–/–^ littermates are defective in RIPK3-mediated necroptosis, it was previously shown that Ripk3^–/–^ BMDMs showed unaltered *C. rodentium*-induced PCD and cytokine responses and that Ripk3^–/–^ mice showed similar *C. rodentium* colonization as WT mice [16]. Therefore, differential *C. rodentium* responses in Casp8^+/-^Ripk3^–/–^ and Casp8^–/–^Ripk3^–/–^ conditions likely reflect caspase-8 mediated signaling effects. Upon analyzing cell membrane permeability in BMDMs we found that Casp8^–/–^Ripk3^–/–^ macrophages showed a small albeit not statistically significant reduction in *C. rodentium*-induced PCD when compared to Casp8^+/-^Ripk3^–/–^ BMDMs (Fig. 5A). In addition, measuring cytokine release showed that *C. rodentium*-infected Casp8^–/–^Ripk3^–/–^ macrophages displayed a trend towards less IL-1β as well as IL-6 secretion (Fig. 5B). We next evaluated caspase-8 activation in *C. rodentium*-infected mice by Western blotting analyses, which showed the p43 and p18 cleaved caspase-8 (cCasp8) fragments in whole colon lysates at 7 dpi (Fig. 5C). Subsequent IHC analyses confirmed the appearance of cCasp8^+^ cells at 7 dpi, and showed that many of these cells were observed deep inside the lamina propria of *C. rodentium*-infected colons (Fig. 5D). In line with this observation, Casp8^–/–^Ripk3^–/–^ mice did not show differences in the numbers of TUNEL^+^ cells or cells with cleaved caspase-3 (cCasp3) when compared to Casp8^+/-^Ripk3^–/–^ mice, as these dying cells were observed mainly at the colonic epithelial border of *C. rodentium*-infected mice (Fig. 5E, F). Importantly, the appearance of cCasp3^+^ cells in *C. rodentium*-infected Casp8^+/-^Ripk3^–/–^ and Casp8^–/–^Ripk3^–/–^ mice was not a consequence of their inability to undergo necroptosis due to RIPK3-deficiency, as caspase-3 cleavage and cCasp3^+^ IECs were observed also in *C. rodentium*-infected WT mice (Fig. S1A, B). Moreover, similar numbers of cCasp3^+^ IECs in *C. rodentium*-infected Gsdmd^–/–^ or Mlkl^–/–^ mice suggested that this cCasp3-staining was indicative of apoptosis (Fig. S1B). Collectively, our observations indicated that *C. rodentium* induces cCasp3-associated but caspase-8-independent apoptosis in IECs.

**Fig. 6:**
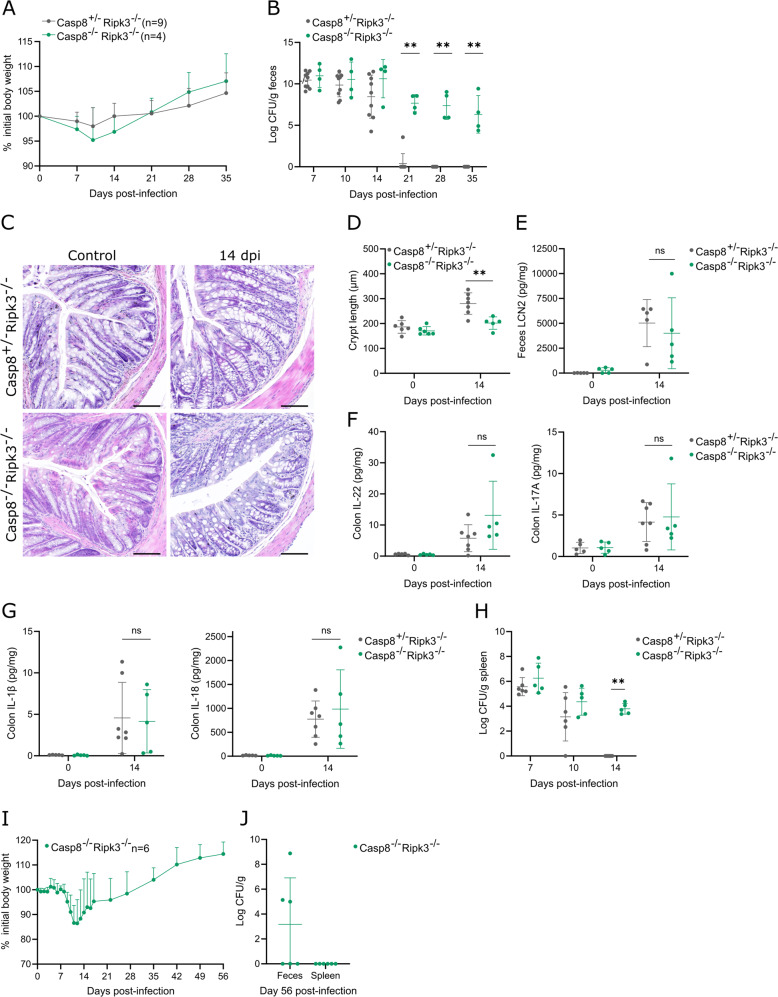
Casp8^–/–^Ripk3^–/–^ mice show impaired intestinal pathogen clearance but maintain systemic host defense against *C. rodentium*. Age- and sex-matched Casp8^+/-^Ripk3^–/–^ (*n* = 9) and Casp8^–/–^Ripk3^–/–^ (*n* = 4) littermates were infected by oral gavage with 5 × 10^9^ CFU *C. rodentium*. **A** Weight change, and **B** fecal *C. rodentium* loads at indicated dpi are shown. Data in (**A**) represent means + SD; data in (**B**) represent individual mice and their means ± SD. Age- and sex-matched Casp8^+/-^Ripk3^–/–^ (*n* = 5–7) and Casp8^–/–^Ripk3^–/–^ (*n* = 5) littermates were infected by oral gavage with 5 × 10^9^ CFU *C. rodentium* and were sacrificed at 14 dpi, along with non-infected control Casp8^+/-^Ripk3^–/–^ (*n* = 6) and Casp8^–/–^Ripk3^–/–^ (*n* = 6) littermates. **C** Representative colon H&E stainings and **D** colon crypt length measurements; **E** fecal LCN2 levels; **F** colon IL-22 and IL-17 levels; and **G** colon IL-1β and IL-18 levels. All data points in (**D**–**G**) represent individual mice along with means ± SD. Scale bars (**C**) 100 µm. **H** Age- and sex-matched Casp8^+/-^Ripk3^–/–^ and Casp8^–/–^Ripk3^–/–^ littermates were infected by oral gavage with 5 × 10^9^ CFU *C. rodentium* and were sacrificed to measure splenic *C. rodentium* loads at 7 dpi (*n* = 5–6), 10 dpi (n = 5-6) or 14 dpi (*n* = 5–7). All data points represent individual mice along with means ± SD. Age- and sex-matched Casp8^–/–^Ripk3^–/–^ littermates (*n* = 6) were infected by oral gavage with 5 × 10^9^ CFU *C. rodentium*. **I** Weight change was monitored and **J** mice were sacrificed at 56 dpi to measure fecal and splenic *C. rodentium* loads. Data in (**I**) represent means + SD; data in (**J**) represent individual mice and their means ± SD.

We next gavaged Casp8^+/-^Ripk3^–/–^ and Casp8^–/–^Ripk3^–/–^ cohorts with *C. rodentium* to evaluate the role of caspase-8 during an in vivo infection. Although both cohorts displayed similar weight changes (Fig. 6A), Casp8^–/–^Ripk3^–/–^ mice continued shedding pathogens in their feces until 35 dpi, one week after their Casp8^+/-^Ripk3^–/–^ littermates had already cleared the infection (Fig. 6B). We next evaluated whether the failure of Casp8^–/–^Ripk3^–/–^ mice to resolve an intestinal *C. rodentium* infection was associated with altered colitis severity at 14 dpi. Interestingly, *C. rodentium*-infected Casp8^–/–^Ripk3^–/–^ colons displayed less crypt hyperplasia than their Casp8^+/-^Ripk3^–/–^ littermates at 14 dpi (Fig. 6C, D). In contrast, caspase-8 did not mediate *C. rodentium*-induced colon inflammation at 14 dpi, as fecal LCN-2 levels, as well as colon cytokine levels, were similar between Casp8^–/–^Ripk3^–/–^ and Casp8^+/-^Ripk3^–/–^ littermates (Fig. 6E–G). Finally, we assessed how inadequate intestinal *C. rodentium* clearance in Casp8^–/–^Ripk3^–/–^ mice impacted on its systemic presence by quantifying splenic pathogen loads at distinct stages of the infection. At 7 dpi similar pathogen numbers were observed in spleens of Casp8^–/–^Ripk3^–/–^ and Casp8^+/-^Ripk3^–/–^ littermates, but the latter cleared the splenic *C. rodentium* loads by 14 dpi while the former remained colonized (Fig. 6H). We then tracked a cohort of *C. rodentium*-infected Casp8^–/–^Ripk3^–/–^ mice until 56 dpi to evaluate potential long-term effects of *C. rodentium* infection in these mice. However, these *C. rodentium*-infected Casp8^–/–^Ripk3^–/–^ mice retained normal body weights and showed no obvious signs of morbidity throughout the experiment (Fig. 6I). Moreover, while half of these mice still displayed fecal shedding at 56 dpi, no pathogens could be detected in their spleens (Fig. 6J). In conclusion, our results in *C. rodentium*-infected Casp8^–/–^Ripk3^–/–^ mice showed that caspase-8 is required to clear intestinal pathogen loads but is not needed for systemic host defense during a chronic intestinal *C. rodentium* infection.

### GSDMD-independent canonical inflammasome responses mediate systemic *C. rodentium* clearance and ensure survival of Casp8^–/–^Ripk3^–/–^ mice

**Fig. 7:**
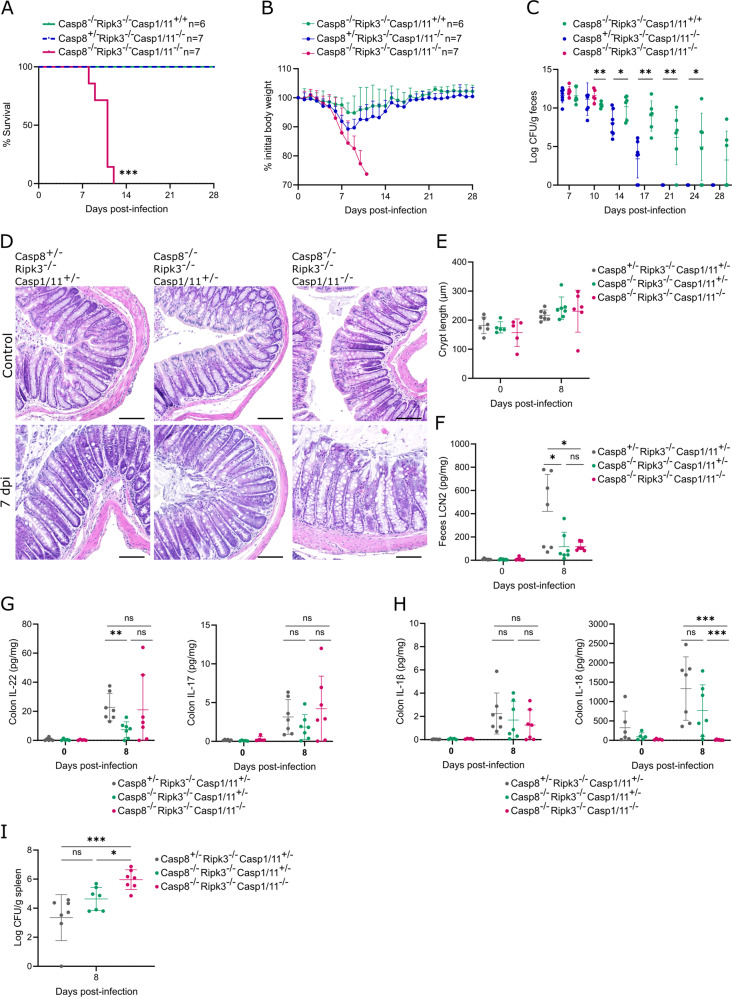
Caspase-1/11 inflammasome responses mediate systemic host defense of Casp8^–/–^Ripk3^–/–^ mice against *C. rodentium* infection. Age- and sex-matched Casp8^–/–^Ripk3^–/–^Casp1/11^+/+^ (*n* = 6), Casp8^+/-^Ripk3^–/–^Casp1/11^–/–^ (*n* = 7) and Casp8^–/–^Ripk3^–/–^Casp1/11^–/–^ (*n* = 7) littermates were infected by oral gavage with 5 × 10^9^ CFU *C. rodentium*. **A** Survival, **B** weight change, and **C** fecal *C. rodentium* loads at indicated days post-infection are shown. Data in (**B**) represent means + SD; data in (**C**) represent individual mice and their means ± SD. Age- and sex-matched Casp8^+/-^Ripk3^–/–^Casp1/11^+/-^ (*n* = 7), Casp8^–/–^Ripk3^–/–^Casp1/11^+/-^ (*n* = 7) and Casp8^–/–^Ripk3^–/–^Casp1/11^–/–^ (*n* = 7) littermates were infected by oral gavage with 5 × 10^9^ CFU *C. rodentium* and were sacrificed at 8 dpi, along with non-infected control Casp8^+/-^Ripk3^–/–^Casp1/11^+/-^ (*n* = 6), Casp8^–/–^Ripk3^–/–^Casp1/11^+/-^ (*n* = 5) and Casp8^–/–^Ripk3^–/–^Casp1/11^–/–^ (*n* = 5) littermates. **D** Representative colon H&E stainings and **E** colon crypt length measurements; **F** fecal LCN2 levels; **G** colon IL-22 and IL-17 levels; **H** colon IL-1β and IL-18 levels; and **I** splenic *C. rodentium* loads. All data points in (**E**–**I**) represent individual mice along with means ± SD. Scale bars (**D**) 100 µm.

Since Casp8^–/–^Ripk3^–/–^ mice retained systemic host defense against *C. rodentium*, we evaluated whether inflammasome-mediated pyroptosis took part in this residual *C. rodentium* resistance. To disable both canonical and non-canonical inflammasome signaling to pyroptosis we crossbred Casp8^–/–^Ripk3^–/–^ mice with mice lacking both caspase-1 and -11. We then subjected the resulting Casp8^–/–^Ripk3^–/–^Casp1/11^–/–^ mice as well as their Casp8^+/-^Ripk3^–/–^Casp1/11^–/–^ and Casp8^–/–^Ripk3^–/–^Casp1/11^+/+^ littermates to a gastrointestinal *C. rodentium* infection. Strikingly, *C. rodentium*-infected Casp8^–/–^Ripk3^–/–^Casp1/11^–/–^ mice showed severe body weight loss and died within 12 dpi, while the other cohorts survived the infection (Fig. 7A, B). Consistent with prior observations, Casp8^–/–^Ripk3^–/–^Casp1/11^+/+^ mice displayed chronic fecal pathogen shedding (Fig. 7C). In contrast, Casp8^+/-^Ripk3^–/–^Casp1/11^–/–^ mice disabled for necroptosis and pyroptosis cleared the infection with normal kinetics (Fig. 7C), in line with the ability of Gsdmd^–/–^Mlkl^–/–^ mice to clear intestinal *C. rodentium* loads.

Given the lethality phenotype of Casp8^–/–^Ripk3^–/–^Casp1/11^–/–^ mice, we next investigated colitis at 8 dpi in these mice as well as in their Casp8^+/-^Ripk3^–/–^Casp1/11^+/-^ and Casp8^–/–^Ripk3^–/–^Casp1/11^+/-^ littermates to evaluate potential cumulative effects of Casp8 and Casp1/11 deletion on a Ripk3^–/–^ background. Although *C. rodentium*-infected mice did not show substantial crypt elongation yet at 8 dpi, no differences could be detected between the different genotypes (Fig. 7D, E). Interestingly, both Casp8^–/–^Ripk3^–/–^Casp1/11^+/-^ and Casp8^–/–^Ripk3^–/–^Casp1/11^–/–^ mice showed diminished fecal LCN2 levels at 8 dpi when compared to Casp8^+/-^Ripk3^–/–^Casp1/11^+/-^ littermates (Fig. 7F). Given that LCN2 is produced mainly by neutrophils [25] we evaluated neutrophil recruitment in these *C. rodentium*-infected mice. However, neutrophils were present in similar numbers in infected colons of Casp8^–/–^Ripk3^–/–^Casp1/11^+/-^ and Casp8^–/–^Ripk3^–/–^Casp1/11^–/–^ mice as compared to Casp8^+/-^Ripk3^–/–^Casp1/11^+/-^ littermates (Fig S2). In addition, at 8 dpi Casp8^–/–^Ripk3^–/–^Casp1/11^+/-^ mice showed less colonic IL-22 production when compared to Casp8^+/-^Ripk3^–/–^Casp1/11^+/-^ littermates (Fig. 7G), which we had not observed in the 14 dpi analyses in Casp8^–/–^Ripk3^–/–^ mice (Fig. 6F). This illustrates the dynamic nature of inflammatory responses during *C. rodentium* infection, as additional IL-22 analyses in Casp8^–/–^Ripk3^–/–^ mice at 7 dpi revealed that these mice produced less IL-22 than their Casp8^+/-^Ripk3^–/–^ littermates at this stage of the infection (Fig S3). These observations suggested that reduced LCN2 as well as IL-22 production may in part explain the reduced ability of caspase-8-deficient mice to clear intestinal pathogen loads. However, fecal LCN2 levels as well as colon IL-22 and IL-17A levels were not further decreased in Casp8^–/–^Ripk3^–/–^Casp1/11^–/–^ mice as compared to Casp8^–/–^Ripk3^–/–^Casp1/11^+/-^ mice, indicating that these features were not mediated by inflammasome responses (Fig. 7F, G). In contrast, we observed that *C. rodentium*-infected Casp8^–/–^Ripk3^–/–^Casp1/11^–/–^ mice showed upregulated IL-1β levels but were incapable of producing IL-18 in the colon (Fig. 7H). Finally, in line with their lethality, Casp8^–/–^Ripk3^–/–^Casp1/11^–/–^ mice showed increased splenic *C. rodentium* loads at 8 dpi when compared to other cohorts (Fig. 7I). Thus, our observations in *C. rodentium*-infected Casp8^–/–^Ripk3^–/–^Casp1/11^–/–^ mice showed that inflammasome responses were responsible for producing IL-18 in the colon, for restraining splenic pathogen loads and eventually for protecting Casp8^–/–^Ripk3^–/–^ mice from lethality.

**Fig. 8:**
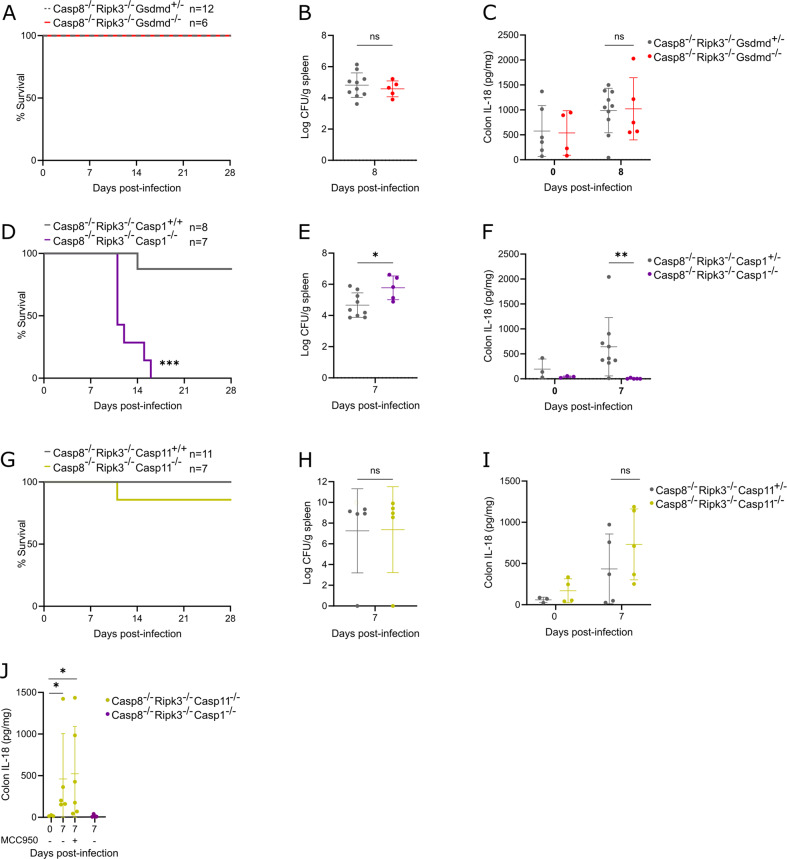
GSDMD-independent canonical caspase-1 inflammasome responses mediate systemic host defense of Casp8^–/–^Ripk3^–/–^ mice against *C. rodentium* infection. **A** Survival analysis of age- and sex-matched Casp8^–/–^Ripk3^–/–^Gsdmd^+/-^ (*n* = 12) and Casp8^–/–^Ripk3^–/–^Gsdmd^–/–^ (*n* = 6) littermates infected by oral gavage with 5 × 10^9^ CFU *C. rodentium*. **B**, **C** Age- and sex-matched Casp8^–/–^Ripk3^–/–^Gsdmd^+/-^ (*n* = 10) and Casp8^–/–^Ripk3^–/–^Gsdmd^–/–^ (*n* = 5) littermates were infected by oral gavage with 5 × 10^9^ CFU *C. rodentium* and were sacrificed at 8 dpi, along with non-infected control Casp8^–/–^Ripk3^–/–^Gsdmd^+/-^ (*n* = 6) and Casp8^–/–^Ripk3^–/–^Gsdmd^–/–^ (*n* = 4) littermates. **B** Splenic *C. rodentium* loads and **C** colon IL-18 levels. All data points in (**B**, **C**) represent individual mice along with means ± SD. **D** Survival analysis of age- and sex-matched Casp8^–/–^Ripk3^–/–^Casp1^+/+^ (*n* = 8) and Casp8^–/–^Ripk3^–/–^Casp1^–/–^ (*n* = 7) littermates infected by oral gavage with 5 × 10^9^ CFU *C. rodentium*. **E**, **F** Age- and sex-matched Casp8^–/–^Ripk3^–/–^Casp1^+/-^ (*n* = 9) and Casp8^–/–^Ripk3^–/–^Casp1^–/–^ (*n* = 5) littermates were infected by oral gavage with 5 × 10^9^ CFU *C. rodentium* and were sacrificed at 7 dpi, along with non-infected control Casp8^–/–^Ripk3^–/–^Casp1^+/-^ (*n* = 3) and Casp8^–/–^Ripk3^–/–^Casp1^–/–^ (*n* = 3) littermates. **E** Splenic *C. rodentium* loads and **F** colon IL-18 levels. All data points in (**E**, **F**) represent individual mice along with means ± SD. **G** Survival analysis of age- and sex-matched Casp8^–/–^Ripk3^–/–^Casp11^+/+^ (*n* = 11) and Casp8^–/–^Ripk3^–/–^Casp11^–/–^ (*n* = 7) littermates infected by oral gavage with 5 × 10^9^ CFU *C. rodentium*. **H**, **I** Age- and sex-matched Casp8^–/–^Ripk3^–/–^Casp11^+/-^ (*n* = 5) and Casp8^–/–^Ripk3^–/–^Casp11^–/–^ (*n* = 5) littermates were infected by oral gavage with 5 × 10^9^ CFU *C. rodentium* and were sacrificed at 7 dpi, along with non-infected control Casp8^–/–^Ripk3^–/–^Casp11^+/-^ (*n* = 3) and Casp8^–/–^Ripk3^–/–^Casp11^–/–^ (*n* = 4) littermates. **H** Splenic *C. rodentium* loads and **I** colon IL-18 levels. All data points in (H-I) represent individual mice along with means ± SD. **J** Colon IL-18 levels of non-infected control Casp8^–/–^Ripk3^–/–^Casp11^–/–^ (*n* = 3) mice as well as Casp8^–/–^Ripk3^–/–^Casp11^–/–^ (*n* = 11) and Casp8^–/–^Ripk3^–/–^Casp1^–/–^ (*n* = 4) mice infected by oral gavage with 5 × 10^9^ CFU *C. rodentium* and sacrificed at 7 dpi. Among the infected Casp8^–/–^Ripk3^–/–^Casp11^–/–^ mice, one cohort was injected daily with vehicle (*n* = 5) and one cohort was injected daily with 50 mg/kg MCC950 (*n* = 6). Data points represent individual mice along with means ± SD.

Next, we aimed to refine the molecular signaling mechanisms by which inflammasomes provide Casp8^–/–^Ripk3^–/–^ mice with these features of host defense against *C. rodentium*. We first investigated the involvement of GSDMD-dependent pyroptosis by generating Casp8^–/–^Ripk3^–/–^Gsdmd^–/–^ and Casp8^–/–^Ripk3^–/–^Gsdmd^+/-^ cohorts that we infected with *C. rodentium*. Intriguingly, ablating Gsdmd on a Casp8^–/–^Ripk3^–/–^ background did not reproduce the *C. rodentium* lethality phenotype as obtained after Casp1/11 deletion, as all Casp8^–/–^Ripk3^–/–^Gsdmd^–/–^ mice survived the infection (Fig. 8A). Accordingly, also the Casp1/11-mediated effects on splenic pathogen loads and colonic IL-18 production were not mediated by GSDMD. Indeed, Casp8^–/–^Ripk3^–/–^Gsdmd^–/–^ mice did not show elevated *C. rodentium* loads in the spleen when compared to Casp8^–/–^Ripk3^–/–^Gsdmd^+/-^ littermates (Fig. 8B), and did show IL-18 upregulation in the colon (Fig. 8C). In line with our prior observations in *C. rodentium*-infected Gsdmd^–/–^ mice, the intact IL-18 production in Casp8^–/–^Ripk3^–/–^Gsdmd^–/–^ colons argued against an involvement of non-canonical inflammasome signaling in which GSDMD acts upstream of inflammasome-mediated cytokine responses. We therefore evaluated whether either canonical caspase-1-mediated or non-canonical caspase-11-mediated inflammasome responses established host defense in Casp8^–/–^Ripk3^–/–^ mice by infecting Casp8^–/–^Ripk3^–/–^Casp1^–/–^ or Casp8^–/–^Ripk3^–/–^Casp11^–/–^ mice with *C. rodentium* (Fig. 8D–I). Strikingly, deleting only caspase-1 in Casp8^–/–^Ripk3^–/–^ mice was sufficient to reproduce the *C. rodentium* lethality phenotype as previously observed in Casp8^–/–^Ripk3^–/–^Casp1/11^–/–^ mice (Fig. 8D). Moreover, Casp8^–/–^Ripk3^–/–^Casp1^–/–^ mice displayed increased pathogen presence in the spleen (Fig. 8E) and were incapable of mounting an IL-18 response in the colon during *C. rodentium* infection (Fig. 8F). In sharp contrast, Casp8^–/–^Ripk3^–/–^Casp11^–/–^ mice survived a *C. rodentium* infection and did not show differences in splenic pathogen loads or colonic IL-18 production at 7 dpi as compared to their *C. rodentium*-infected Casp8^–/–^Ripk3^–/–^Casp11^+/-^ littermates (Fig. 8G–I). Together, these results show that systemic host defense of Casp8^–/–^Ripk3^–/–^ mice against the non-canonical inflammasome activator *C. rodentium* fully relied on GSDMD-independent canonical caspase-1 mediated inflammasome responses. Finally, to investigate whether canonical Nlrp3 inflammasome responses were responsible for inducing colonic IL-18 production, we treated Casp8^–/–^Ripk3^–/–^Casp11^–/–^ mice that are only capable of activating canonical inflammasomes with the Nlrp3 inhibitor MCC950 during *C. rodentium* infection. Interestingly, while this experiment confirmed that canonical inflammasome deficient Casp8^–/–^Ripk3^–/–^Casp1^–/–^ mice did not produce IL-18 at 7 dpi, daily MCC950 treatment was not capable of reducing IL-18 production in the colon of Casp8^–/–^Ripk3^–/–^Casp11^–/–^ mice at 7 dpi (Fig. 8J). This observation suggests that instead of Nlrp3 the NLRC4 canonical inflammasome that was previously implicated in *C. rodentium* pathogenesis [30] might be responsible for *C. rodentium*-induced colonic IL-18 production in Casp8^–/–^Ripk3^–/–^ mice. However, further research will be needed to formally investigate the roles of Nlrp3 and NLRC4 in host defense of Casp8^–/–^Ripk3^–/–^ mice against *C. rodentium* infection.

## Discussion

The existence of distinct signaling pathways for executing cellular suicide via either apoptosis, necroptosis or pyroptosis is a crucial aspect of host defense against pathogens [1, 2]. Indeed, studies using cell death deficient genetic mouse models illustrated that several types of PCD need to be disabled to render the host susceptible to an infection. For instance, it was shown that both Casp1/11^–/–^ and Casp8^–/–^Ripk3^–/–^ mice resisted an intravenous *Salmonella* Typhimurium infection, while Casp8^–/–^Ripk3^–/–^Casp1/11^–/–^ mice succumbed to this infection [31]. This in vivo *S*. Typhimurium susceptibility correlated with the inability to undergo PCD, as only Casp8^–/–^Ripk3^–/–^Casp1/11^–/–^ macrophages fully resisted *S*. *Typhimurium*-induced PCD [31]. Similarly, in contrast to its cytotoxic effect in Ripk3^–/–^Casp1/11^–/–^ macrophages, *Legionella pneumophila* could not induce PCD in Casp8^–/–^Ripk3^–/–^Casp1/11^–/–^ macrophages and therefore achieved higher intracellular replication in these cells [32]. These studies using intracellular pathogens revealed a direct correlation between the ability to kill infected host cells through redundant PCD modes and in vivo resistance to that pathogen, aligning with the view that host cell death destroys the replicative niche of intracellular pathogens and exposes them to the host’s immune system for clearance [1, 2]. Compared to intracellular bacterial pathogens, the role of PCD during in vivo infections with extracellular bacterial pathogens appears to be more complex. For instance, GSDMD has been reported as either beneficial or detrimental during infections with extracellular pathogens. Indeed, Gsdmd^–/–^ mice were shown to be more susceptible to an oral infection with *Yersinia pseudotuberculosis* [33], while they were protected from an intraperitoneal infection with *E. coli* [34]. Likewise, Casp8^–/–^Ripk3^–/–^ mice were shown to be susceptible to a subcutaneous *Yersinia pestis* infection but resistant against an intratracheal infection with *E. coli* [35, 36]. Although the reasons underlying these complex and apparently contradictory observations are unclear, the in vivo role of a particular PCD mode may depend on the capacity of the invading extracellular pathogen to trigger and modulate host signaling pathways as well as on its route of administration.

In this study, we aimed to delineate the roles of pyroptosis, necroptosis and apoptosis in host defense against *C. rodentium* administered through its natural gastrointestinal route. Quite surprisingly, our observations indicated that these three PCD modes were largely dispensable for host defense against this extracellular enteropathogen. Regarding pyroptosis and necroptosis, Gsdmd^–/–^ mice, Mlkl^–/–^ mice as well as Gsdmd^–/–^Mlkl^–/–^ mice cleared the infection with normal kinetics, showing that neither GSDMD-mediated pyroptosis nor MLKL-mediated necroptosis were essential for host defense against *C. rodentium*. Nevertheless, despite not showing altered numbers of dying IECs, Gsdmd^–/–^ mice displayed a transient increase in fecal *C. rodentium* loads around 14 dpi. Interestingly, IEC-specific GSDMD-deficient mice were shown to display a similar *C. rodentium* susceptibility without effects on epithelial cell death [37]. Instead, impaired mucus layer formation was suggested to explain *C. rodentium* susceptibility of these GSDMD^IEC-KO^ mice [37], raising the possibility that the increased *C. rodentium* loads in Gsdmd^–/–^ mice at 14 dpi could be explained by a mucus defect. Alternatively, we cannot exclude that cytotoxic functions of GSDMD in macrophages or neutrophils play a role in the *C. rodentium* susceptibility of Gsdmd^–/–^ mice. GSDMD was shown to mediate NETosis in *C. rodentium*-infected human neutrophils [38], and NETosis-defective PAD4^–/–^ mice show a similarly moderate *C. rodentium* susceptibility phenotype in the colon [39]. Therefore, a role for GSDMD-mediated NETosis remains possible in local intestinal host defenses against *C. rodentium*. Regardless, given the moderate effects of GSDMD deficiency during *C. rodentium* infection, it should be mentioned that other Gasdermins might compensate for the absence of GSDMD. For instance, GSDMA or GSDME might contribute to *C. rodentium* host defense, as both of these Gasdermins were shown to be activated and to contribute to cell death upon other bacterial infections [40–43]. In addition, GSDME was shown to exert inflammatory effects in mouse models of colon inflammation [44, 45], suggesting GSDME as a particularly suitable candidate for future cell death studies in *C. rodentium* infection. Ultimately, future research will be needed to fully understand the cell type specific functions of GSDMD as well as the compensatory roles of other Gasdermins during a gastrointestinal *C. rodentium* infection.

Regarding apoptosis, the survival of Casp8^–/–^Ripk3^–/–^ mice upon *C. rodentium* infection showed that caspase-8-mediated apoptosis did not synergize with RIPK3-dependent necroptosis for systemic host defense against this enteropathogen. In contrast, caspase-8 contributed to intestinal defense against *C. rodentium*, as Casp8^–/–^Ripk3^–/–^ mice displayed chronic intestinal *C. rodentium* colonization. However, *C. rodentium*-infected Casp8^–/–^Ripk3^–/–^ mice did not display diminished epithelial cCasp3^+^ cells. This suggests that the intestinal host defense function of caspase-8 signaling does not reside in IECs, consistent with the observed location of cCasp8^+^ cells inside the lamina propria of *C. rodentium* infected mice. In addition, although apoptotic signaling in these cCasp8^+^ cells is possible, their location suggests that protective caspase-8 signaling during *C. rodentium* infection may rather act in immune cells in which it can induce pro-inflammatory and anti-microbial gene expression [36, 46–48]. In addition, this transcriptional stimulatory function of caspase-8 was suggested to facilitate *C. rodentium*-induced inflammasome activation in cultured macrophages [16], suggesting that perhaps part of its role in intestinal host defense could be mediated by inflammasome signaling. Finally, since *C. rodentium*-induced cCasp3^+^ apoptosis was not driven by caspase-8-mediated apoptosis, IECs in *C. rodentium*-infected mice may die through the mitochondrial caspase-9-mediated intrinsic apoptosis pathway. Several attaching and effacing pathogens such as *C. rodentium* modulate intrinsic apoptosis both in negative and in positive manners [4], but a possible role of this PCD mode in host defense against *C. rodentium* will need to be addressed in future studies.

A remarkable finding in our study is the fact that host defense against *C. rodentium*, which is a prototypical activator of caspase-11-dependent non-canonical inflammasome-mediated pyroptosis [17–20], crucially relied on GSDMD-independent canonical inflammasomes. Indeed, *C. rodentium* infection of Casp8^–/–^Ripk3^–/–^Casp1^–/–^ mice recapitulated the infection-induced lethality of Casp8^–/–^Ripk3^–/–^Casp1/11^–/–^ mice, while both Casp8^–/–^Ripk3^–/–^Casp11^–/–^ and Casp8^–/–^Ripk3^–/–^Gsdmd^–/–^ mice survived the infection. This observation that caspase-1 but not caspase-11 was responsible for protecting Casp8^–/–^Ripk3^–/–^ mice against *C. rodentium* is in line with infections using NleB- and NleF-mutant pathogens. Although NleF inhibits caspase-11, NleF deletion had no additive effect in attenuated NleB mutants that cannot inhibit death receptor induced apoptosis and necroptosis [5–7, 10, 11]. Therefore, these bacterial genetic studies indicated that non-canonical inflammasome signaling did not cooperate with apoptotic and necroptotic signaling to fight *C. rodentium* infections. Nevertheless, the caspase-1-mediated canonical inflammasome pathway that confers systemic host defense against *C. rodentium* remains unclear. Although the Nlrp3 inflammasome was proposed to combat *C. rodentium* [49], our observation that pharmacological Nlrp3 inhibition in Casp8^–/–^Ripk3^–/–^Casp11^–/–^ mice that are deficient for non-canonical inflammasome signaling did not impair colonic IL-18 production suggests that residual canonical Nlrp3 signaling is not responsible for this response during *C. rodentium* infection. As such, the NLRC4 inflammasome could be a plausible candidate for triggering canonical caspase-1 protective effects, since its activation in non-hematopoietic cells was suggested to participate in host defense against *C. rodentium* [30]. In addition, epithelial NLRC4 activation is known to trigger intestinal IL-18 production in mice [50, 51]. However, despite the clear correlation between the survival of *C. rodentium*-infected Casp8^–/–^Ripk3^–/–^ mice with additional Casp1/11, Casp1, Casp11 or GSDMD deficiencies and their ability to produce colonic IL-18, we do not know whether driving IL-18 production is a crucial host defense function of canonical caspase-1 signaling in Casp8^–/–^Ripk3^–/–^ mice. Indeed, the role of IL-18 during a gastrointestinal *C. rodentium* infection appears to be context-dependent. One study reported that up to 40% of IL-18 deficient mice succumbed to a *C. rodentium* infection [52], but two other studies observed that disabling IL-18 production or signaling rendered mice only slightly more susceptible to the infection [15, 49], and yet another study found no differences in the capacity of IL-18^–/–^ mice to cope with *C. rodentium* [53]. Therefore, more research is required to identify the upstream activator of canonical inflammasome activation as well as to evaluate the role of downstream IL-18 production in host defense during *C. rodentium* infection.

Taken together, in this study we performed a systematic side-by-side comparison of the separate as well as the additive effects of various cell death signaling proteins responsible for executing distinct modes of PCD during gastrointestinal *C. rodentium* infection. We revealed a crucial cooperation between caspase-8 signaling and GSDMD-independent canonical caspase-1 signaling to establish full host defense against gastrointestinal *C. rodentium* infection, which sets the stage for further research to elucidate the upstream triggers and downstream mediators executing these host defense functions.

## Materials and methods

### Mice

The Gsdmd^–/–^ [21], Casp1/11^−/−^ [54], Casp1^−/−^ [55], Casp11^–/–^ [17], Mlkl^−/−^ [56], and C8^–/–^Ripk3^–/–^ [28, 29, 57, 58] genetic mouse models used in this study, either generated on C57BL/6J background or backcrossed at least ten generations to C57BL/6J background, were described previously. More complex genetic mouse models used in this study were generated by intercrossing the above lines. All mice used in in vivo infection experiments were age- and sex-matched littermates. All mice were housed in individually ventilated cages in the specific pathogen-free animal facility of the IRC-VIB. Food and water were provided ad libitum. All animal experiments were performed according to institutionally approved protocols according to national (Belgian Laws 14/08/1986 and 22/12/2003, Belgian Royal Decree 06/04/2010) and European (EU Directives 2010/63/EU, 86/609/EEG) animal regulations. Animal protocols were reviewed and approved by the Ethical Committee Animal Experimentation VIB site Ghent—Ghent University—Faculty of Sciences (permit number LA1400091) with approval ID 2019-072. All necessary efforts were made to minimize suffering of the animals.

### *C. rodentium* infection of macrophages

Primary macrophages were generated from bone marrow cells flushed from femur and tibia. Cells were differentiated to bone marrow-derived macrophages (BMDMs) by culturing them in Iscove’s modified Dulbecco’s medium (IMDM, Lonza) with 10% (v/v) heat-inactivated FBS, 30% (v/v) L929 cell-conditioned medium, 1% Gibco non-essential amino acids, penicillin (100 U/ml) and streptomycin (100 mg/ml) for 6 days in 37 °C in the presence of 5% CO_2_. After differentiation, medium was aspirated and the cells were scraped in IMDM supplemented with 10% FBS and 1% Gibco non-essential amino acids. For specific experiments, 6.5 × 10^4^ cells or 4.5 × 10^5^ cells were seeded per well in respectively 96-well or 24-well plates and were incubated overnight at 37 °C and 5% CO_2_. On day 7, *C. rodentium* infections were performed with the nalidixic acid (NAL) resistant ICC169 strain at the logarithmic phase of proliferation. For *C. rodentium* infections LPS-primed or unprimed BMDMs were used. LPS priming was performed by aspirating the medium and adding medium containing 500 ng/ml LPS for 4 h. BMDMs were infected with either live *C. rodentium* (MOI10) or provided with equal amounts of heat-killed (95 °C, 10 min) *C. rodentium*. At 2 h post-infection 100 µg/ml gentamycin was added. The cells were incubated for 24 h post-infection at 37 °C and 5% CO_2_ before sample collection.

### Cell death analysis

BMDMs were seeded in 96-well plates (6.5 × 10^4^ cells/well) and were infected with either live *C. rodentium* (MOI10) or provided with equal amounts of heat-killed (95 °C, 10 min) *C. rodentium* the next day. Cellular membrane integrity was determined with an IncuCyte FLR imaging system (Essen Bioscience), using the non-cell-permeable SYTOX Green (SG) DNA staining agent (250 nM) (Invitrogen) according to the manufacturer’s protocol. Two hours post-infection gentamycin (100 μg/ml) was added to the cells. Each hour an image was obtained with a minimum of two image fields per well. The percentage of SG-positive cells was calculated with the IncuCyte software package. These percentages were normalized to a 100% dead cell count control achieved by SG labeling of Triton X-100 treated wells. Cell death measurements were conducted with biological triplicates, using technical duplicates for each experimental condition.

### Gastrointestinal *C. rodentium* infection

Age- and sex-matched mice were infected by oral gavage with 5 × 10^9^ CFU of the nalidixic acid (NAL) resistant ICC169 *C. rodentium* strain administered in a 200 μl inoculum in the logarithmic phase of proliferation, as described [59]. Overall susceptibility to the infection was evaluated by survival, weight loss and fecal and systemic bacterial loads. Enumeration of *C. rodentium* over the course of the infection was performed by plating stool samples and spleen tissues collected at the indicated time points after infection, on selective Luria-Bertani (LB) agar containing 50 μg/ml NAL. Colony forming units (CFUs) were normalized to the weight of the sample. Mice that did not surpass the threshold of 1 × 10^5^ CFU/g feces at 7 dpi were considered not successfully infected and were removed from the experiment. To inhibit the NLRP3 inflammasome mice were injected intraperitoneally daily with 50 mg/kg MCC950 (MCE MedChemExpress, HY-12815A) or with vehicle.

### Lipocalin-2 ELISA

After enumeration of fecal *C. rodentium* loads, fecal samples were further cleared upon full-speed centrifugation for 30 min at 4 °C. Fecal lipocalin-2 levels in the supernatant were then analyzed using the mouse Lipocalin-2/NGAL duoset ELISA (R&D systems) according to the manufacturer’s instructions and were normalized per mg of feces.

### Cytokine measurements

Tissue samples were weighed and were homogenized in 500 μl PBS with protease inhibitors, after which lysis was completed by addition of lysis buffer (20 mM Tris HCl (pH 7.4), 200 mM NaCl, 1% Nonidet P-40) and incubation for 20 min on ice. Full-speed centrifugation for 30 minutes cleared the homogenate and supernatant was used for further analysis. Mouse cytokines in cell culture supernatants and tissue homogenates were determined by magnetic bead-based multiplex assay using Luminex technology (Bio-Rad, Hercules, CA, USA) according to the manufacturer’s instructions. Cytokines from tissue homogenates were normalized to weight of tissue, while cytokines from cell culture supernatants were expressed as concentration per ml of cell culture medium.

### Histology

Colon tissues were fixed in 4% paraformaldehyde, embedded in paraffin, and cut into 4 µm sections. For histopathological analysis hematoxylin and eosin staining were performed according to standard protocols. Crypt lengths were measured using Image-J-win4. Cell death was evaluated on paraffin sections by TUNEL staining (in situ cell death detection kit, TMR red, Roche) performed according to the manufacturer’s instructions. For immunohistochemical staining paraffin sections were rehydrated and except for the F4/80 staining heat-induced antigen retrieval was performed in Antigen Unmasking Solution, Citric Acid Based (Vector Laboratories). Endogenous peroxidase activity was blocked by incubating the slides in 3% H_2_O_2_ (Sigma). The blocking buffer contained 0.2% goat serum, 0.5% fish skin gelatin and 2% BSA in PBS for anti-cCasp8 and anti-cCasp3, 1% BSA in PBS for anti-GSDMD, 5% BSA in PBS for Ly6G and 5% NGS and 1% BSA in PBS for F4/80. Primary antibodies for IHC were anti-cCasp3 Asp175 (Cell Signaling, 9661S), anti-cCasp8 (Asp387) (D5B2) (Cell signaling, 8592S), GSDMD (Abcam, ab219800) [60], F4/80 (Biorad, MCA497G) or Ly6G (BD Biosciences, 551459). Biotinylated secondary antibodies were purchased from Dako (E0432), BD pharmagen (559286) and vector labs (BA-4001). Stainings were visualized with Vectastain ELITE ABC Kit, Peroxidase (Standard) (Vector Laboratories) and DAB substrate (ImmPACT DAB Substrate kit, Peroxidase, Vector Laboratories). Incubation times with DAB substrate were equal for all samples, after which sections were counterstained with hematoxylin and imaged. All pictures were taken with a high-content screening microscope (Zeiss AxioScan) at the same exposure and intensity settings. Subsequently, positive cells per cross-section were counted manually, or in case of F4/80 staining areas were quantified using QuPath software. All histological crypt length and cell number quantifications were performed in a blinded fashion.

### Western Blot analysis

Cells and culture supernatants, or whole colon homogenates, were incubated with cell lysis buffer (20 mM Tris HCl (pH 7.4), 200 mM NaCl, 1% Nonidet P-40), and denatured in Laemlli buffer by boiling for 10 min. Proteins were separated by SDS-PAGE electrophoresis (Thermo Scientific) after which proteins were transferred to membranes using turbo (7 min) blotting. Blocking and antibody incubation were performed in PBS supplemented with 0.05% Tween20 (vol/vol) and 3% non-fat dry milk. The membranes were incubated overnight at 4˚ C with primary antibodies against caspase-1 (1:1000; Adipogen, AG-20B-0042-C100), IL-1β (1:2000; GeneTex, GTX74034), GSDMD (1:1000, Abcam, ab209845), anti-cCasp3 Asp175 (1:1000, Cell Signaling, 9661S), caspase-3 (1:1000, Cell signaling, 9662S), cleaved-caspase-8 (Asp387) (D5B2) (1:1000, 8592S, Cell signaling), MLKL (1:1000, Sigma-Aldrich, MABC604) or pMLKL (Ser345) (D6E3G) (1:1000, Cell signaling, 37333). After washing, membranes were incubated with HRP-conjugated anti-mouse, anti-rabbit or anti-rat antibodies (1:5000; Jackson ImmunoResearch Laboratories, 115-035-146, 111-035-144 and 112-035-143) or were incubated with the directly labeled primary antibody β-actin-HRP (1:10000; Santa Cruz) for up to 3 h. Proteins of interest were detected by the enhanced SuperSignal West Pico Chemiluminescent Substrate (Thermo Scientific).

### Statistics

All statistical analyses were performed using Graphpad Prism version 9.0. For mouse survival curves, statistical significance was determined by log-rank Mantel-Cox test. Other data were analyzed by applying either unpaired two-sided student *t*-tests or unpaired two-sided Mann-Whitney tests in case of not normal distribution of the values. Data are shown as means of biological replicates with SD as indicated in figure legends. Statistical results are indicated as ns not significant; *p < 0.05; **p < 0.01 or ***p < 0.001.

## Supplementary information

Supplementary information

Original Data File

Reproducibility checklist

## Data Availability

All data generated and analyzed during this study are included in this published article and its supplementary information files.
