# Supplementary material for: Gasdermin D independent canonical inflammasome responses cooperate with caspase-8 to establish host defense against gastrointestinal Citrobacter rodentium infection
Source: Cell Death Dis. 2023 Apr 21;14(4):282. doi: 10.1038/s41419-023-05801-4 (PMC10119323; doi:10.1038/s41419-023-05801-4)
Supplement: Supplementary file 1 — Supplementary information [file 41419_2023_5801_MOESM1_ESM.docx]

**Gasdermin D independent canonical inflammasome responses cooperate with caspase-8 to establish host defense against gastrointestinal *Citrobacter rodentium* infection**

Elien Eeckhout^1,2^, Lisa Hamerlinck^1,2^, Veronique Jonckheere^3^, Petra Van Damme^3^, Geert van Loo^2,4^, Andy Wullaert^1,2,5,#^

*^1^Department of Internal Medicine and Paediatrics, Ghent University, 9052 Ghent, Belgium;*

*^2^VIB-UGent Center for Inflammation Research, VIB, 9052 Ghent, Belgium;*

*^3^iRIP unit, Laboratory of Microbiology, Department of Biochemistry and Microbiology, Ghent University, Ghent, Belgium;*

*^4^Department of Biomedical Molecular Biology, Ghent University, 9052 Ghent, Belgium;*

*^5^Laboratory of Proteinscience, Proteomics and Epigenetic Signalling (PPES), Department of Biomedical Sciences, University of Antwerp, Antwerp, Belgium*

**Supplementary Figures**


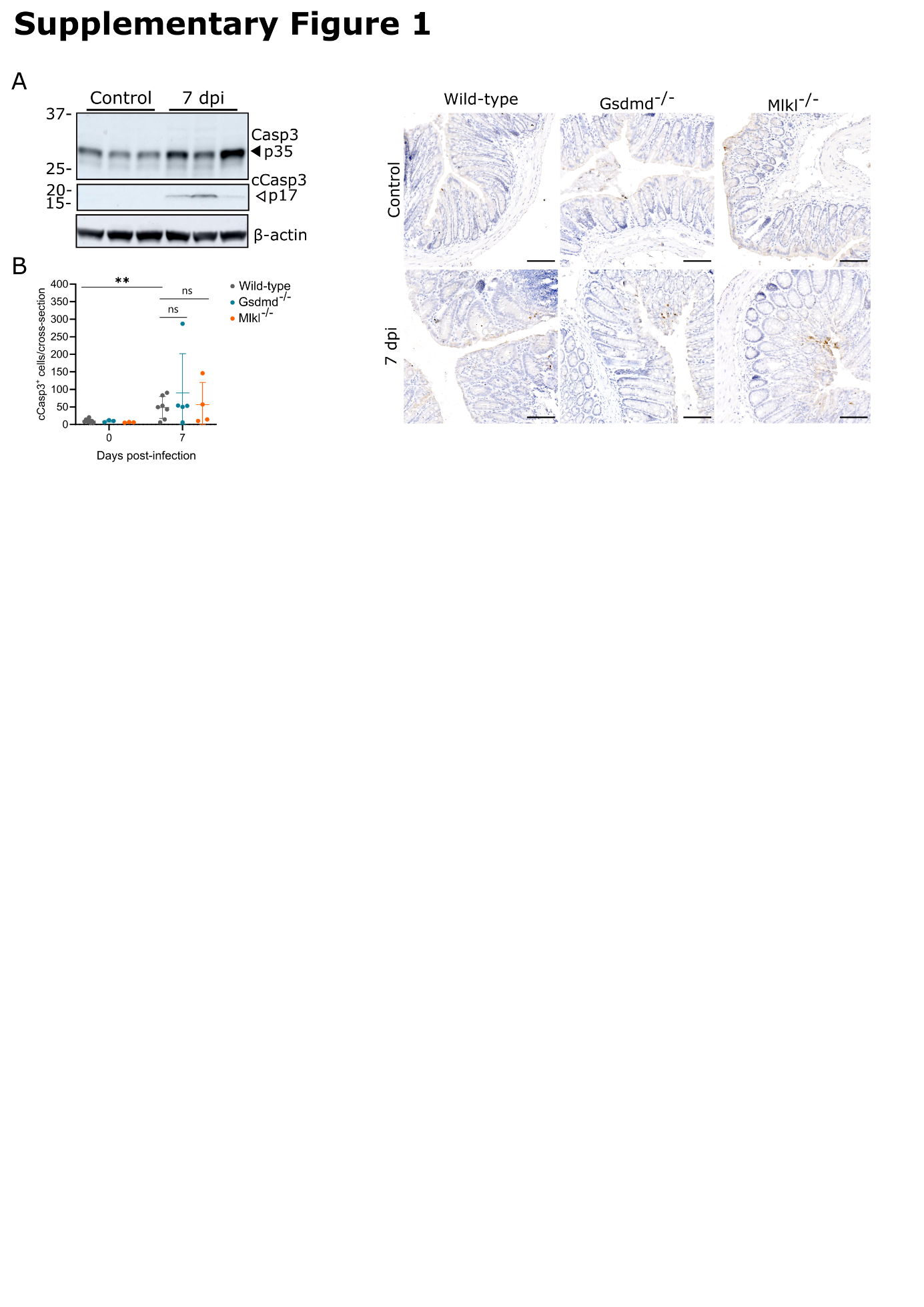


**Supplementary Figure 1. Caspase-3 cleavage is observed in WT mice upon gastrointestinal *C. rodentium* infection and is not diminished in *C. rodentium*-infected Gsdmd^-/-^ or Mlkl^-/-^ mice.** (A) Western blot analyses for full length caspase-3 (p35) and cCasp3 (p17) on whole colon lysates from WT mice not infected or infected with 5x10^9^ CFU *C. rodentium* for 7 days. Every lane represents a whole colon lysate from a different mouse. (B) Representative colon cCasp3 IHC stainings and quantifications from wild-type, Gsdmd^-/-^ and Mlkl^-/-^ mice not infected or infected with 5x10^9^ CFU *C. rodentium* for 7 days. Every data point in the quantification represents a different mouse with means +/- SD, n=3-8 per group. Scale bars 100µm.


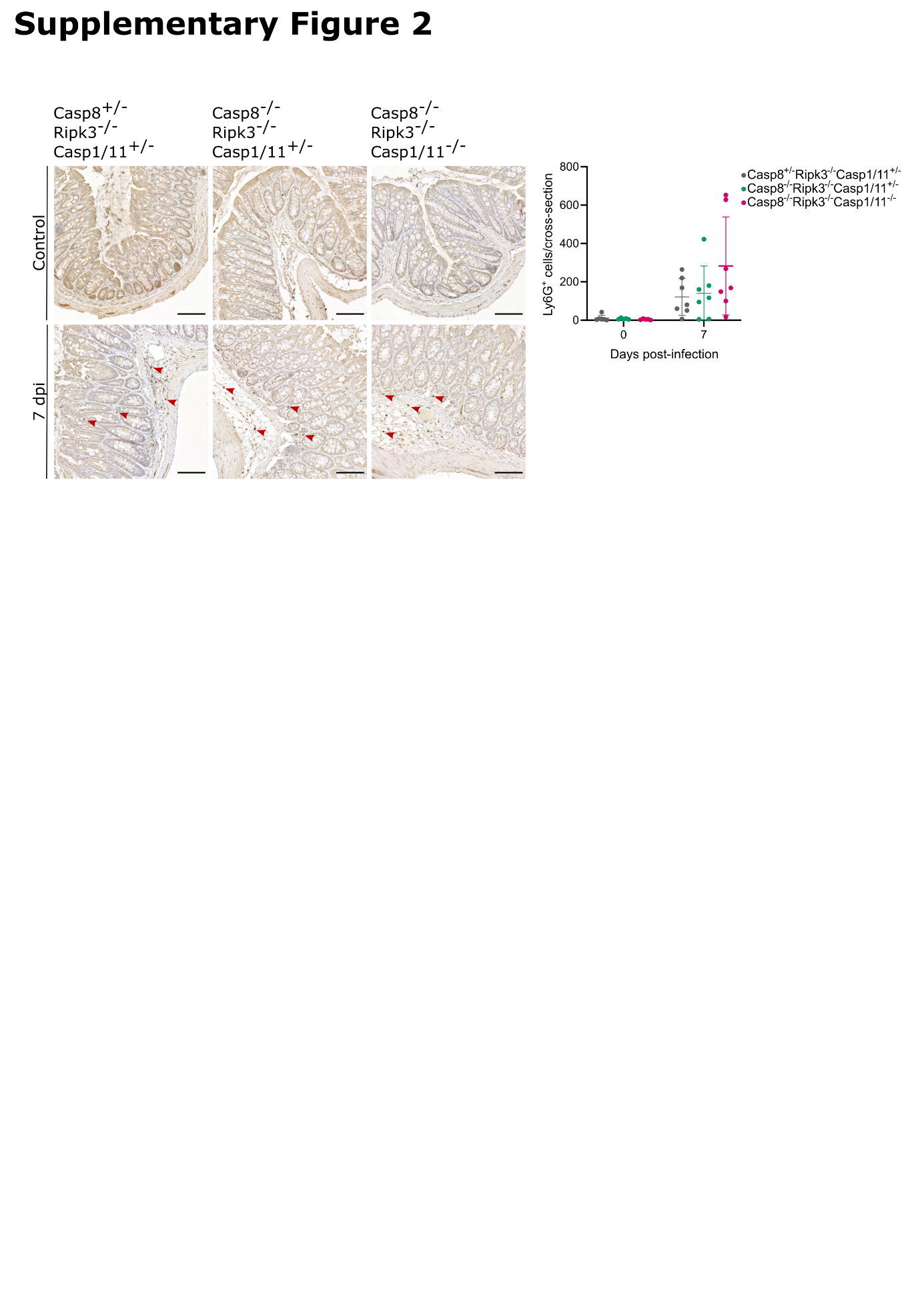


**Supplementary Figure 2. Caspase-8 deficiency is not associated with impaired neutrophil recruitment upon *C. rodentium* infection at 8 dpi.**

Age- and sex-matched Casp8^+/-^Ripk3^-/-^Casp1/11^+/-^ (n=7), Casp8^-/-^Ripk3^-/-^Casp1/11^+/-^ (n=7) and Casp8^-/-^Ripk3^-/-^Casp1/11^-/-^ (n=7) littermates were infected by oral gavage with 5x10^9^ CFU *C. rodentium* and were sacrificed at 8 dpi, along with non-infected control Casp8^+/-^Ripk3^-/-^Casp1/11^+/-^ (n=6), Casp8^-/-^Ripk3^-/-^Casp1/11^+/-^ (n=5) and Casp8^-/-^Ripk3^-/-^Casp1/11^-/-^ (n=5) littermates. Representative IHC stainings as well as quantification are shown for Ly6G^+^ neutrophils (some examples indicated with red arrowheads) in the colon. Scale bars 100µm.


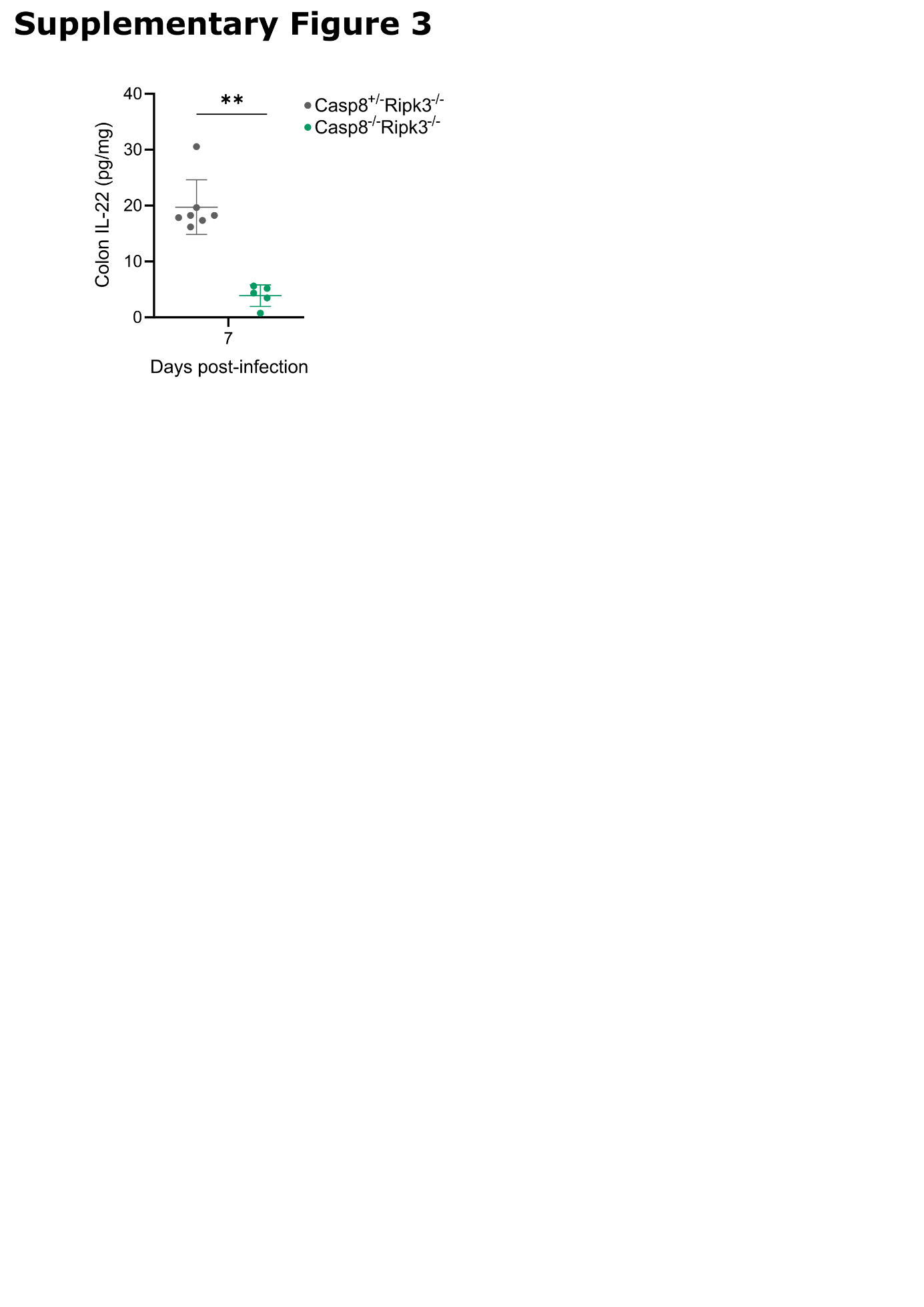


**Supplementary Figure 3. Casp8^-/-^Ripk3^-/-^ mice show impaired colonic IL-22 production at 7 dpi during *C. rodentium* infection.**

Colon IL-22 levels form age- and sex-matched Casp8^+/-^Ripk3^-/-^ (n=7) and Casp8^-/-^Ripk3^-/-^ (n=5) littermates that were infected by oral gavage with 5x10^9^ CFU *C. rodentium* and were sacrificed at 7 dpi. Data points represent individual mice along with means +/- SD.
