# Supplementary material for: Gasdermin D independent canonical inflammasome responses cooperate with caspase-8 to establish host defense against gastrointestinal Citrobacter rodentium infection
Source: Cell Death Dis. 2023 Apr 21;14(4):282. doi: 10.1038/s41419-023-05801-4 (PMC10119323; doi:10.1038/s41419-023-05801-4)

**Gasdermin D independent canonical inflammasome responses cooperate with caspase-8 to establish host defense against gastrointestinal *Citrobacter rodentium* infection**

Elien Eeckhout^1,2^, Lisa Hamerlinck^1,2^, Veronique Jonckheere^3^, Petra Van Damme^3^, Geert van Loo^2,4^, Andy Wullaert^1,2,5,#^

*^1^Department of Internal Medicine and Paediatrics, Ghent University, 9052 Ghent, Belgium;*

*^2^VIB-UGent Center for Inflammation Research, VIB, 9052 Ghent, Belgium;*

*^3^iRIP unit, Laboratory of Microbiology, Department of Biochemistry and Microbiology, Ghent University, Ghent, Belgium;*

*^4^Department of Biomedical Molecular Biology, Ghent University, 9052 Ghent, Belgium;*

*^5^Laboratory of Protein Chemistry, Proteomics and Epigenetic Signalling (PPES), Department of Biomedical Sciences, University of Antwerp, Antwerp, Belgium*

**Raw data**


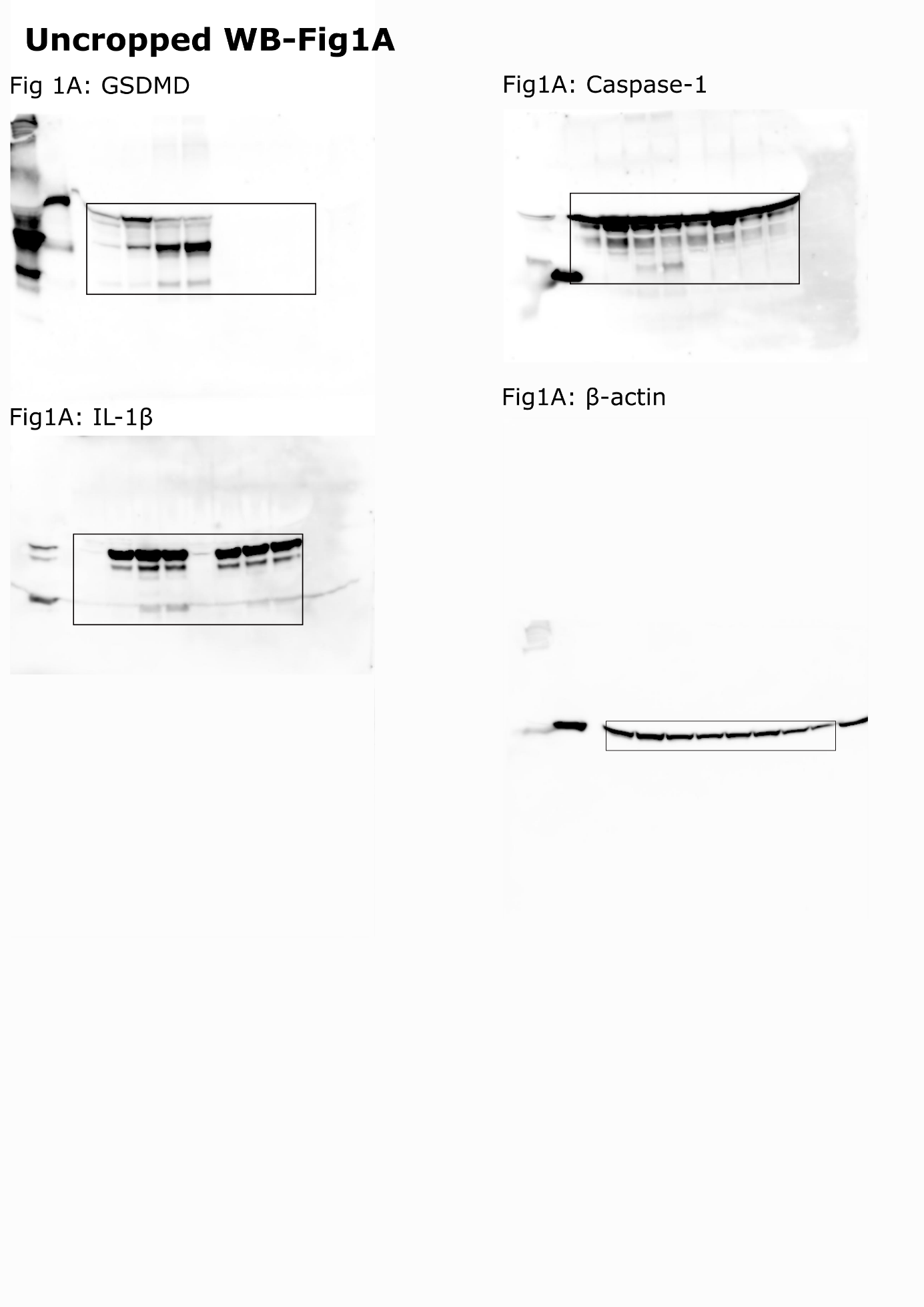


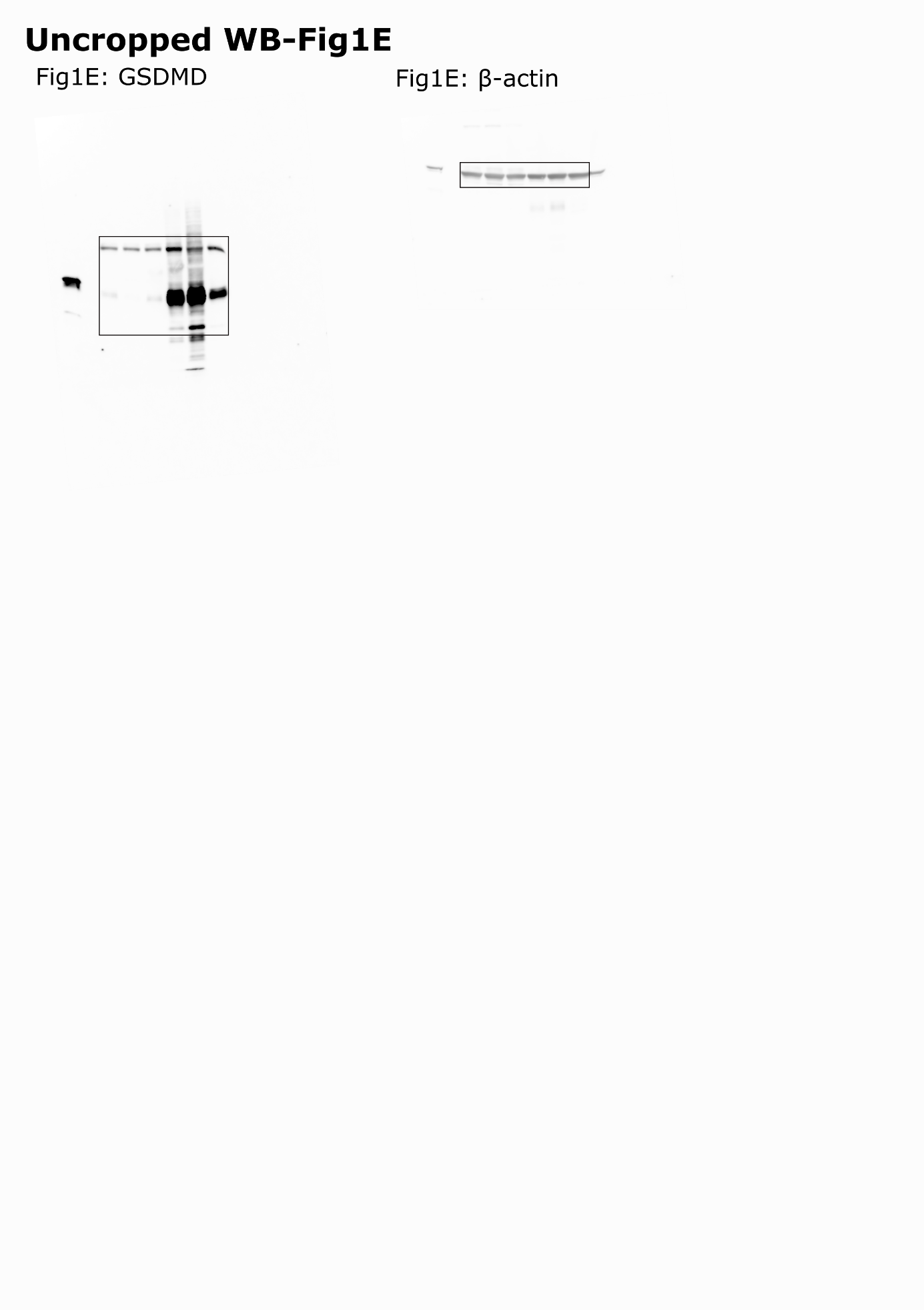


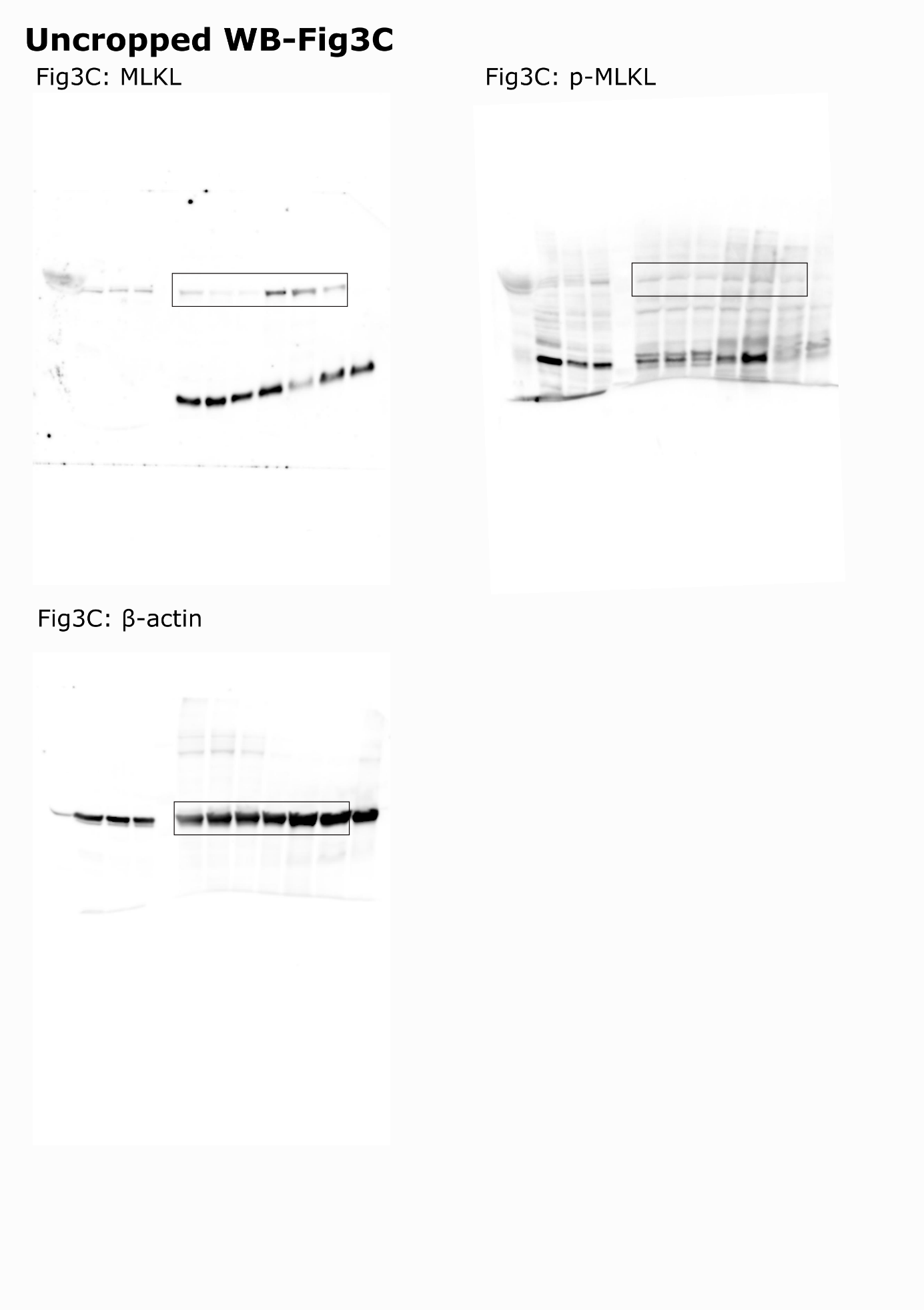


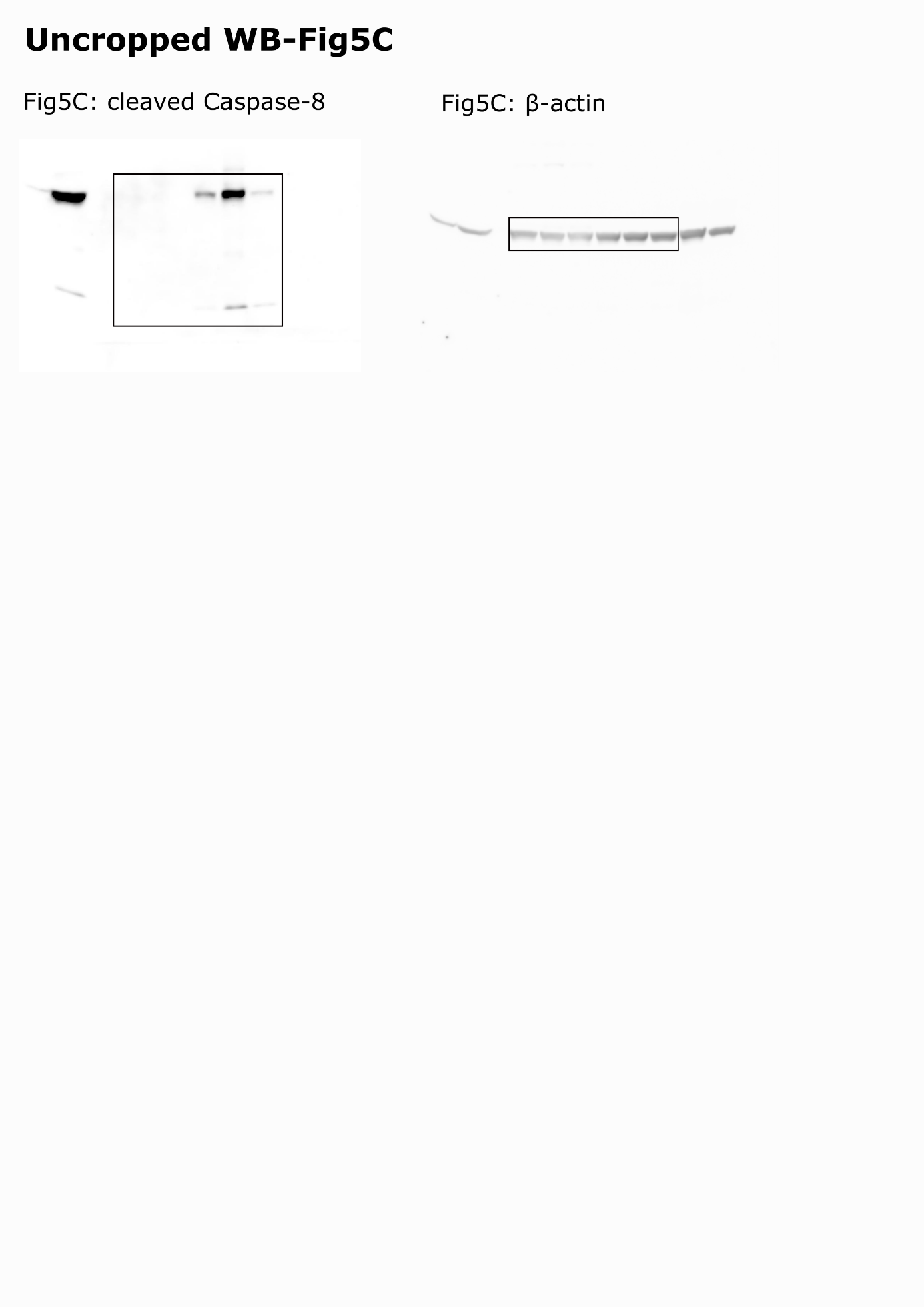


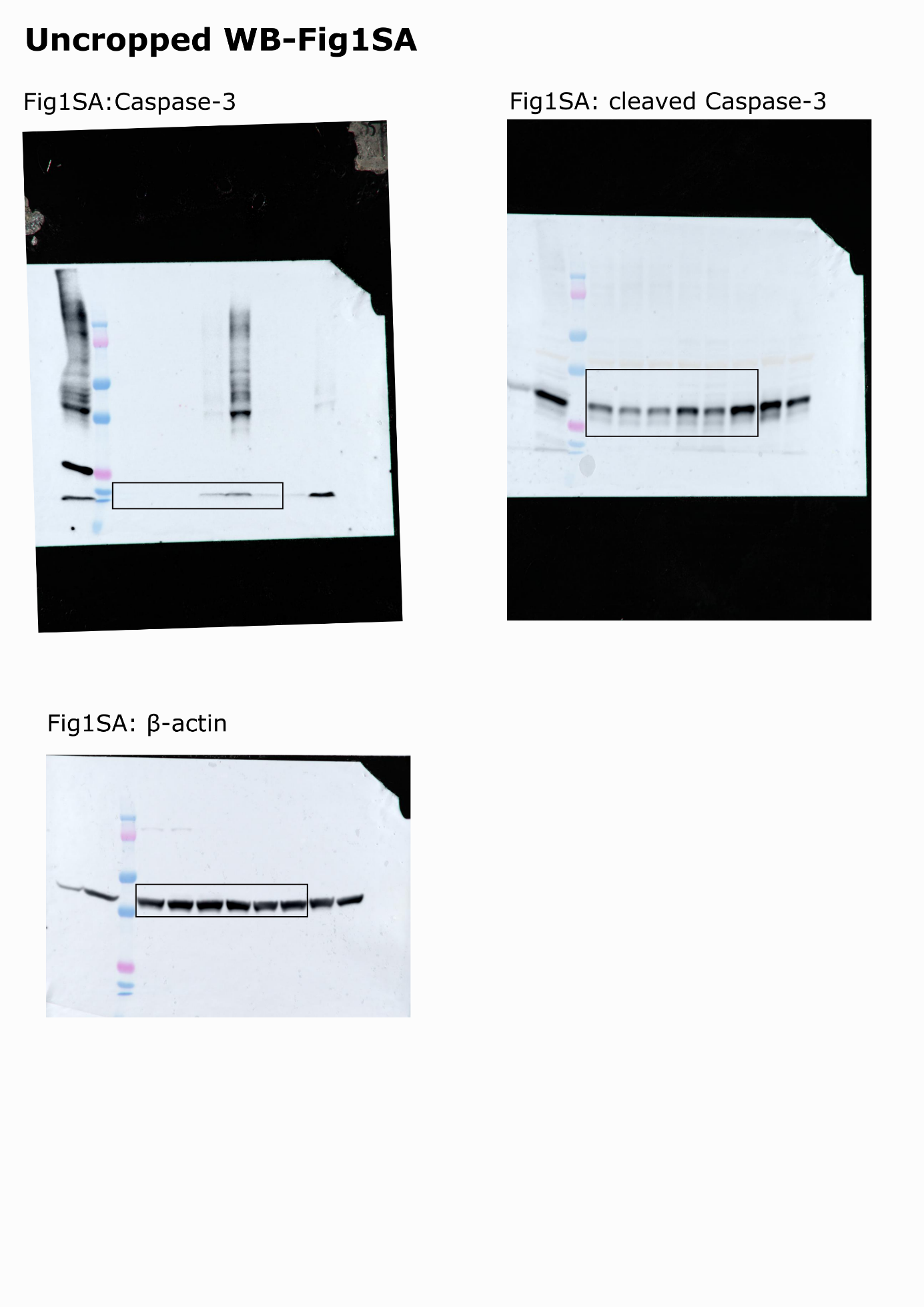

Supplement: Supplementary file 2 — Original Data File [file 41419_2023_5801_MOESM2_ESM.docx]
